# Antibiotic Use in Agriculture and Its Consequential Resistance in Environmental Sources: Potential Public Health Implications

**DOI:** 10.3390/molecules23040795

**Published:** 2018-03-30

**Authors:** Christy Manyi-Loh, Sampson Mamphweli, Edson Meyer, Anthony Okoh

**Affiliations:** 1 Fort Hare Institute of Technology, University of Fort Hare, Alice Campus, Alice 5700, Eastern Cape, South Africa; smamphweli@ufh.ac.za (S.M.); emeyer@ufh.ac.za (E.M.); 2 Applied and Environmental Microbiology Research Group (AEMREG), Department of Biochemistry and Microbiology, University of Fort Hare, Alice Campus, Alice 5700, Eastern Cape, South Africa; aokoh@ufh.ac.za; 3 SAMRC Microbial Water Quality Monitoring Centre, University of Fort Hare, Alice Campus, Alice 5700, Eastern Cape, South Africa

**Keywords:** antibiotics, agriculture, antibiotic resistance, environmental sources, developing countries

## Abstract

Due to the increased demand of animal protein in developing countries, intensive farming is instigated, which results in antibiotic residues in animal-derived products, and eventually, antibiotic resistance. Antibiotic resistance is of great public health concern because the antibiotic-resistant bacteria associated with the animals may be pathogenic to humans, easily transmitted to humans via food chains, and widely disseminated in the environment via animal wastes. These may cause complicated, untreatable, and prolonged infections in humans, leading to higher healthcare cost and sometimes death. In the said countries, antibiotic resistance is so complex and difficult, due to irrational use of antibiotics both in the clinical and agriculture settings, low socioeconomic status, poor sanitation and hygienic status, as well as that zoonotic bacterial pathogens are not regularly cultured, and their resistance to commonly used antibiotics are scarcely investigated (poor surveillance systems). The challenges that follow are of local, national, regional, and international dimensions, as there are no geographic boundaries to impede the spread of antibiotic resistance. In addition, the information assembled in this study through a thorough review of published findings, emphasized the presence of antibiotics in animal-derived products and the phenomenon of multidrug resistance in environmental samples. This therefore calls for strengthening of regulations that direct antibiotic manufacture, distribution, dispensing, and prescription, hence fostering antibiotic stewardship. Joint collaboration across the world with international bodies is needed to assist the developing countries to implement good surveillance of antibiotic use and antibiotic resistance.

## 1. Introduction

The term antibiotics encompass a wide range of chemical substances that are produced naturally, semi-synthetically, and synthetically, and are used to inhibit (bacteriostatic) bacterial growth or kill them (bactericidal) [1,2,3]. They are categorized based on their effects as either bacteriostatic or bactericidal, and on their series of efficacy, as narrow or broad-spectrum antibiotics. Furthermore, the classes of drugs that are more widely used in agriculture at the global level, which are of growing scientific concern with regards to their potential adverse effects and risk management steps, include the tetracyclines, aminoglycosides, β-lactams, lincosamides, macrolides, pleuromutilins, and sulphonamides [4,5,6,7]. Gelband et al. [8] noted that these antibiotics have the same mode of actions or belong to the same general classes as those used for humans; a situation that demands the judicious use of these drugs in animal farming, as there is bound to be a degree of interaction between animals and humans.

Markedly, the antibiotic consumption patterns in agriculture vary across regions and countries in the developing world, and even antibiotics that have been banned in other countries, including the developed countries, are still being used in most developing countries [9,10]. However, the antibiotic consumption profiles in developing countries are greatly influenced by the gross abuse and misuse of antibiotics due to their availability over the counter, through unregulated supply chains as well as the purchase without prescriptions [11]. Also, Van Boeckel et al. [12] projected that the antibiotic consumption will approximately double in the BRICS countries consisting of Brazil, Russia, India, China, and South Africa. The forecast is propelled by a shift to large-scale farms requiring the routine use of antibiotics to maintain the health of animals and productivity. The shift is caused by the progress in consumer demand for animal products. Resistance to antibiotics is an inherent side effect associated with the overuse, abuse, or substantial use of antibiotics [13,14].

The antibiotic resistance pattern varies between regions and countries corresponding to the degree of antibiotic consumption, which is guided and regulated by the antibiotic policies of a particular country [15,16]. Nevertheless, China has been registered as the world’s leading producer and consumer of both animals and human antibiotics. Antibiotic-related crisis is ascribed to the misuse of antibiotics that are, ultimately, discharged into the environment, the presence of antibiotic residues (parent antibiotic or its metabolites or both found in animal derived products) in livestock products and wastes, and lastly, the lack of stringent and effective supervision and control over antibiotics production, use, and disposal [17]. Human activities in response to industrialization drastically heightened the availability of antibiotic residues in food and the environment, and the development and distribution of antibiotic resistant bacteria along with their resistance genes, thus causing an increase in the abundance of resistant bacteria and genes [4].

The antibiotic residues, and antibiotic-resistant bacteria and resistance genes are considered as environmental pollutants and responsible for a tenacious public health crisis throughout the globe [18]. The health challenges linked to antibiotic-resistant microorganisms are more about restricted therapeutic remedies in most developing countries that lack access to good quality treatment, thus, accentuating infection as an important root of morbidity and mortality [19]. However, the soil and water environment have been regarded as vital reservoirs and sources of antibiotic resistance [20,21]; more so, as they are affected by agriculture [22]. Not only does the administration of antibiotics in food-producing animals facilitate antibiotic resistance, but it may also result in the presence of antibiotic residues (including the parent compounds or its metabolites, or both) in animal-derived products (muscles, kidney, liver, fat, milk, and egg) available for human consumption. However, these antibiotic residues have been reported to exert a huge and negative impact on public health and food safety with regards to drug toxicity, immunopathological diseases, carcinogenicity, allergic reactions, and drug sensitization, amongst others [7,23,24,25]. These adverse impacts tend to be influenced by land use, contaminated water sources, national policies (that symbolize production, trade, animal health, and food security), national and international trade, animal demography, and interactions between the human populations as well, as they are reported to vary considerably between regions and countries [26].

In a nutshell, antibiotic resistance is observed as a “One Health subject”, both as a cause and solution encompassing the interactions between humans, animals, and the environment [27]. Accordingly, in an attempt to contain antibiotic resistance, the World Health Organisation instituted a Global Action Plan (GAP) which demands that each country should develop national action plans in line with the key actions of the GAP, but with respect to its financial resources and extent of its problems [28]. Surveillance and monitoring of antibiotic use and antibiotic resistance is one facet of the strategies against antibiotic resistance. However, developing countries encounter challenges regarding surveillance systems because of lack of capacity and integration [29].

This paper assembles information about antibiotic and antibiotic resistance in animals, animal-derived products, and the agriculture-impacted environment. Basically, it covers antibiotics used in agriculture, ways through which they end up in the environment causing antibiotic pollution, and on the other hand, the consequential effects of antibiotic residues on public health. In depth, the consequential and devastating effect of antibiotic use, known as antibiotic resistance, has been deliberated on to include salient aspects, such as the determination of antibiotic resistance, antibiotic resistance in livestock farming, as well as antibiotic resistance in manure-impacted environment (soil and water).

## 2. Antibiotics in Agriculture

The use of antibiotics is not only constrained to the clinical settings, as prescriptions involved in the therapeutic regimens for the eradication of diseases in humans. It is also employed in livestock farming, where antibiotics can be used for disease treatment of animals, and in sub-therapeutic levels in concentrated animal feed for growth promotion, improved feed conversion efficiency, and for the prevention of diseases [22,30,31]. Of great concern, the uses, types, and mode of actions of the antibiotics employed in agriculture and veterinary practice are closely related or the same (that may belong to the same general classes, function and act in similar ways) to those prescribed to humans [32]. Clearly, the choice of antibiotics and the antimicrobial consumption pattern demonstrates geographical variation across the continents being influenced by the food animal species, regional production patterns and types of production system, intensive or extensive farming, purpose of farming (commercial or industrial or domestic), lack of clear legislative framework or policies on the use of antibiotics, as well as the size and socioeconomic status of the population, and the farmers in particular [12,33].

The inclusion of nonessential antibiotics in animal feed for growth promotion purposes remains largely unregulated in the underdeveloped countries [34]. The persistent use of these nonessential antibiotics in livestock farming can be attributed to the expansion and greater concentration of farmlands, inadequate governmental policies, and control over the use and sales of antibiotics, reduced use of infection control measures, and the unwillingness of farmers to execute delegated changes in farm practices [35]. Developing countries continue to employ the antimicrobial agent for growth promotion to maintain the healthy state of the animals, to increase productivity, and raise incomes for the farmers [36,37]. However, these are contradictory to the Swedish agricultural data, as it recorded no loss of production after the ban exercise [36].

Altogether, Boeckel et al. [12] noted that on a global scale, the average antimicrobial agent consumed per annum of animal produced (per kg) varied across the animal species with values of 45 mg/kg, 148 mg/kg, and 172 mg/kg associated with cattle, chicken, and pigs, respectively. Equally, their mode of administration differs with the animal types. In this light, Apata [38] noted that antibiotics were added to water and feed for chicken in sub-therapeutic levels for growth promotion and prophylaxis. This had a devastating effect, as even healthy birds were unnecessarily exposed to antibiotics. Moreover, as these birds compete for food sources, eventually, there exists a difference in the doses consumed between the individuals, with one receiving a higher dose than others. This introduces another differential in the selective pressure on commensals, which could lead to the selection of resistant commensals that would eventually end up in the environment [39]. Singer et al. [40] accorded the administration of antibiotics in animal feed or water, in which the animals are reared in groups, making it difficult to isolate only the infected animals, as well as that the isolation process could be stressful to the animals and dangerous to the veterinarian who has to administer the antibiotic process.

Contrarily, Sekyere [41], in their study, demonstrated the administration of antibiotics to pigs via the intravenous route for treatment, and in this case, shunned the exposure of healthy animals to antibiotics. However, this mode of administration might cause the accumulation of these drugs in adipose tissues, thereby posing a health risk to consumers of pork fat. In addition, Cromwell [42] mentioned that varying quantities of antibiotics are being employed at the different stages of livestock production, especially in pig farming, that incorporates four stages viz. gestation, farrowing, weaning, and finishing. Kim et al. [43] emphasized the significant difference in the use of antibiotics amongst piglets, fattening pigs, and sows during therapy and growth promotion; antibiotics are employed in pig farming for treatment, metaphylaxis, prophylaxis, and growth promotion. The authors further recorded a significant difference in the use of antibiotics between the three production systems in poultry farming, including breeding poultry, broilers, and laying hens. Accordingly, these may release different masses of remnant antibiotics into the environment [30].

Generally, in the developing countries, the level and rate of antibiotic utilization in the farming sector might be influenced by the manner in which the farmers acquire (over the counters) and use these antibiotics (multidrug practices), and also, the presence of existing factors. The existing factors include a high prevalence or level of infections, profound scarcity of state management and development strategies, shortfall in husbandry zone planning, negligible hygienic practices in livestock husbandry in conjunction with the presence of an integrated agricultural system [32,44]. Specifically, in Vietnam, there has been reported cases of frequent and uncontrolled epidemic diseases, such as the porcine reproductive and respiratory syndrome (PRRS), foot and mouth disease, and digestive tract infections and reproductive disorders in piglets and exotic sows, respectively. The disease conditions necessitate the wide use of antibiotics by producers in livestock for the prevention and therapy of diseases as one of the most likely approaches to combat diseases [45]. Moreover, the country practices an integrated agriculture–aquaculture farming system, whereby the aquaculture is being sustained via livestock and human wastes. This further strengthens the risk of exposure of humans, animals, and environment to available antibiotics [43].

Similarly, Guetiya Wadoum et al. [25] noted the multidrug practices by farmers in addition to the use of formulations with low doses of antibiotics that do not indicate the active ingredients, or the withdrawal periods in poultry farming, in Cameroon. As a result, they diagnosed the diseases that occurred or threatened the chickens and decided on the types of antibiotics and dosage to employ, as well, the veterinarians even gave wrong diagnosis about the diseases amongst the birds to encourage and promote the sales of their drugs. What a bizarre situation that creates chances for the abuse of antibiotics? Several authors have demonstrated the indiscriminate use of antibiotics by farmers, and attributed it to lack of knowledge on the prudent use of these drugs, and the possible adverse effects associated with their abuse, non-adherence to manufacturer’s instructions, and the antibiotic withdrawal periods, unavailability of veterinarians and their services. Furthermore, inexperienced farmers relied on the knowledge and advice of experienced farmers and local drug sellers for drug administration, and above all, wealthy farmers tended to employ multiple antibiotics, since they have the potential to acquire the drugs. Also, very few animals are referred to the laboratory for diagnosis to identify the causative agent and to assess the antibiotic susceptibility testing prior to antibiotic application [10,32,37,41,46].

As a consequence, some of these underdeveloped countries still employ some antibiotics, such as chloramphenicol, tylosin, and TCN (a powder mixture that consisted of oxytetracycline, chloramphenicol, and neomycin) which have been banned for use in the developed countries. Accordingly, these drugs have been associated with aggravation of kidney disease (neomycin), carcinogenicity, mutagenicity, and development of aplastic anemia in humans (chloramphenicol) [47,48]. In addition, Guetiya Wadoum et al. [25] mentioned that TCN and tylosin had to be withdrawn for 21 days and 10 days respectively, before the sales of eggs or meat; a situation which is quite difficult for the farmers to implement and respect. This expedites the consumption, by humans, of poultry products harbouring antibiotic residues.

Notwithstanding, the available limited data on antimicrobial utilization in livestock farming ensues the partial reports of antimicrobial consumption and sales. This is due to the lack of surveillance systems subsidized by the government to monitor antimicrobial use and resistance, the lack of knowledge and the reluctance of food animal producers, animal feed producers, public health and veterinary officers and veterinary pharmaceutical companies to provide such in-depth measurements [49,50]. In conclusion, the information presented herein is deduced from the findings obtained by several authors who have previously conducted investigations on antibiotic use and antibiotic resistance, yet the rate of antibiotic usage and antibiotic resistance is alarming; imagine the scenario in which the real/actual data has to be presented. Seemingly, there is a need to call for cooperation or team collaboration from individuals, farmers, veterinarians, consumers, and local vendors of pharmaceutical products for the prudent use of antibiotics both in the clinical and agricultural settings across the nations or countries, in a bid to circumvent the rising antibiotic resistance of bacteria [51].

### 2.1. Antibiotics’ Introduction into the Environment

The indiscriminate and abusive use of antibiotics can result in higher concentrations of antibiotics in the environment, which can be termed as antibiotic pollution. The sources via which antibiotics can be released into the environment are diverse, including the human waste streams, and wastes from veterinary use and livestock farming [3]. Antibiotics used for prophylaxis or therapy in humans contaminate the human waste streams, likewise, the antibiotics used in animals for growth promotion, prevention, and treatment equally contaminate the animals’ waste streams. Thus, these are considered as prime sources of antibiotic release into the environment [52]. This is because the administered antibiotics are not fully metabolized, and are released unchanged into the environment, i.e., water, manure or soils. The amount and rate at which the antibiotics are being released into the environments depends on the specific antibiotic and its administered dosage, as well as the species and the age of the animals [51]. Nevertheless, these waste streams will contain both the antibiotics and resistance genes; both considered as pollutants, and their fate in the environment differ [49].

Furthermore, antibiotics and their metabolites contained in stockpiled animal manure may seep through the pile to surface and groundwater, and also into the soil. This is especially so for antibiotics with high water affinity or which are water soluble, thus making their spread and ecotoxicity in the environment faster, and widely with the aid of water fluidity [53]. In the same view, antibiotics can be introduced into the environment via soil fertilization with raw animal manure, irrigation with wastewater generated from farm activities, or via accidental release by runoffs from farms [54]. Interestingly, Hamscher et al. [55] noted that dust contaminated with antibiotics from farms could equally serve as another route of environmental release of these drugs. Chee-Sanford et al. [56] also emphasized the release of antibiotics into the environment via the dispersal of feed and accidental spill of products, as well as discharges.

In addition, Sekyere [41] noted that pig farmers in some different districts in the Ashanti Region of Ghana do not secure their antibiotics, thereby making them freely accessible for use and abuse by unauthorized persons and children. Also, the farmers disposed of their used antibiotic containers by merely throwing them into drains, refuse dumps, or onto bare ground, instead of burying them as recommended. The author further mentioned that these antibiotics were stored under suboptimal environmental conditions, vulnerable to temperature fluctuations that could accelerate their decomposition, thereby causing a reduction in their concentration and efficacy during administration. Such circumstances promote antibiotic resistance of bacteria living in the gastrointestinal tracts of the animals, due to constant exposure to sublethal levels of these antibiotics, or could even cause prompt administration of an overdose of the antibiotics which is noted to be inefficient. More especially, in commercial and intensive poultry farming, antibiotics may be administered to the entire animal population in feed or water, rather than targeting only the diseased animals. Thus, resistance becomes unavoidable [57]. Interestingly, antibiotics produced naturally by environmental microorganisms, to deter competitors from living space and food, are gradually accumulating in the environment [58]. Seemingly, antibiotics are released from their production facilities in high concentrations into the environment [59]. Also, Sahoo and colleagues [60] noted that antibiotics could be found in the natural environment via improper disposal of out-of-date drugs from pharmaceutical shops, and unwanted, expired household pharmaceuticals.

Accordingly, these antibiotics released usually consist of different types, and consequently, they do not degrade, all at the same time, i.e., they degrade at different rates in the environment over time by the main elimination processes, including sorption, photo degradation, biodegradation, and oxidation [61,62]. Albeit, other applied methods, such as adsorption, filtration, coagulation, sedimentation, advanced oxidation processes have been implemented [63]. Specifically, several findings have demonstrated the use of composting, and anaerobic and aerobic digestion to cause the reduction of the antibiotic’s level in manure, wastewater, and sludge, but these processes vary in efficiency with the category of the antibiotics, the conditions employed for composting, as well as the type of livestock manure [53,64]. Nonetheless, the presence of these antibiotics in the environment may create selective pressure resulting in antibiotic resistance and also the removal processes, reduce the concentrations of these antibiotics, allowing time for the exposed bacteria to develop resistance which may be presented as stress adaptation, co-selection, cross-resistance, and cross-protection.

Moreover, the use of antibiotics urges susceptible bacteria to these antibiotics to develop resistance in a bid to survive. In this view, bacteria prevaricate the inhibitory or bactericidal activities of the antibiotics, and execute resistance by either modifying or altering the target sites (ribosomes) for binding by antibiotics, with the help of ribosomal protection proteins which bind to the ribosomes, thereby preventing the binding and interference of protein synthesis [65,66] or neutralizing antibiotics via enzymes produced by adding acetyl or phosphate groups to the precise site on the antibiotics [67], or finally, via changing of membrane permeability due to the presence of efflux pumps on the cell membrane [68,69]. Furthermore, the sensitive bacteria tend to survive in an antibiotic polluted environment by acquiring antibiotic resistance genes from other bacteria or phages (lateral gene transfer), undergo mutations in specific antibiotic gene targets, and by altering of the bacterial surfaces [70].

### 2.2. Animal-Derived Products and Antibiotic Pollution vs Public Health

In developing countries, food prepared and sold by street vendors is in vogue, and it is still emerging hastily in some countries, notably Indonesia, Cameroon, and Democratic Republic of Congo [71,72]. These foods usually comprise of meat (beef, pork, snails) either raw, roasted, or cooked in sauce/stew, starchy foods and snacks, which are sold in restaurants located in public places (markets, schools, hospitals), on the ground in the streets, and along main roads [73]. It is for this reason that foodborne outbreaks are highest in developing countries, and dawdles as an issue of public health concern worldwide, because it is indicated as one of the significant food safety hazards concomitant with animal-derived foods [74]. Cooked foods sold on the street have a great socioeconomic impact; they create jobs and provide income to low or unskilled men and women, as well as serve as a major channel for the supply of food to financially handicapped individuals or poor and less privileged individuals [75]. However, there is increased meat consumption to meet the protein demand of the population [72].

Antibiotics have been reported to accumulate and form residues at varying concentrations in the tissues and organs of food animals, as presented in Table 1. Billah et al. [24] referred to these antibiotic residues as chemical residues or pharmacologically active substances representing either the parent compound or its degraded products, which are released, gathered, or stored in the edible tissues of the animal, due to their use in the prevention, treatment, and control of animal diseases. Undoubtedly, in Cameroon, Guetiya Wadoum et al. [25] demonstrated the presence of chloramphenicol and tetracycline residues in concentrations above the maximum residue limit (MRL) recommended by the European Union in 2010, in edible chicken tissues (muscle, gizzards, heart, liver, kidney) and eggs. Similarly, Billah et al. [24] detected ciprofloxacin in higher concentration in egg white, but in lower concentration in egg yolk during treatment of the birds. Also, Olufemi and Agboola [76] reported a high oxytetracycline residue in edible beef tissues of cattle slaughtered at Akure, in Nigeria, at violating levels beyond the MRL stipulated by WHO. However, of profound concern are circumstances in which diseased animals and animals undergoing therapy could be sold quickly to save funds, or could be slaughtered and used as food or feed for other animals [43]. This causes difficulties in the prophylactic approach to handling epidemic diseases and health risks to consumers, as well as a negative influence on the environment. Van Ryssen [77] reported the use of poultry litter as a feed to farm animals in South Africa, since it is considered as a bulky protein supplement.

Ideally, no animal derived product should be consumed unless there is a complete absence of residual amounts of administered drugs. Nevertheless, the intriguing fact is that there are constant detectable levels of residues, identified via the help of markedly improved analytical methods. Therefore, the world regulatory authorities have set the MRL for various veterinary drugs that should be expected and considered safe in foods for human consumption [78,79]. According to Beyene [80], the diet, age and disease status of the animal added to the absorption, distribution, metabolism, and excretion of the drugs, the extra-label drug use and the improper withdrawal times, amongst others, are the risk factors responsible for the development of residues. In this light, farmers are supposed to adhere and implement the right dosages of the antibiotics, as well as observed their withdrawal periods prior to slaughter and market, in a bid to avoid illegal concentrations of drug residues in the animal products. The withdrawal period (clearance or depletion time) defines the length of time required for an animal to metabolize the administered antibiotics under normal conditions, and also, the time needed for the antibiotic concentration in the tissues to reduce to a safe and acceptable level described as tolerance. It can equally be referred to, the time interval necessary between the last administration of the drug under normal conditions of use to animals and the time when treated animals can be slaughtered to produce foodstuff safe for public consumption. Depending on the drug product, route of administration, and dosage form (even with the same active ingredients), the withdrawal periods vary from a day to several days or weeks, and according to the target animals [81].

It has been reported that the health of humans correlates directly with the environment (i.e., their habitat and its components, including plants, animals, microorganisms, and other human beings) and the quality of food that they consume [60,82]. Taking into consideration the growing human population, the changing standard of living conditions, the food shortages, and the greater demands for the intensified production of animal proteins for human consumption across the globe, essential practices to improve on the agricultural and industrial productivity are needed [83]. Of interest is the critical use of antibiotics in agriculture to meet the demands of the rising human population, as the use of antibiotics in this setting has been associated with several benefits. It is therefore anticipated that, in the future, almost all the animals slaughtered and consumed as food must have received a chemotherapeutic or a prophylactic agent of some sort [81]. However, the consumption of these meats, milk, and eggs contaminated with antibiotic residues usually has tremendous impacts on the health of humans. These effects may be direct or indirect, owing to the high dose of the residues, which must have accrued over a prolonged period [81]. They can be exhibited as drug hypersensitivity reactions, aplastic anemia, carcinogenic, mutagenic, immunologic and teratogenic effects, nephropathy, hepatotoxicity, disruption of the normal flora of the intestines, a reproductive disorder, as well as the development of antibiotic-resistant bacteria in the gut [80,81,84].

## 3. The Great Challenge: Antibiotics Resistance

The routine employment of antibiotics, for prevention and growth promotion purposes in livestock farming, selects for antibiotic resistance among commensal and pathogenic bacteria. Owing to the fact that most of these antibiotics are not fully metabolized but released into the environment as waste products, antibiotic resistance has an ecological impact, since these waste products still have the potential to influence the bacteria population and promote antibiotic resistance. Cogliani et al. [36] pointed out that the low concentrations of these antibiotics in the environment bring about random and spontaneous mutagenesis. Therefore, the environment has been viewed as a plausible reservoir or pool of antibiotics and antibiotic-resistant bacteria, as well as their resistance genes [97]. It is a situation of great concern to public health facilities worldwide, as bacteria have the capability to transfer resistance genes between strains of the same species and between different species [98]. This is, however, possible due to the fact that the antibiotic resistance genes are located on elements, including transposons, integrons, and plasmids, that can be immobilized [99]. The transmission of these resistance genes is termed horizontal gene transfer (HGT) or lateral gene transfer (LGT), and it does occur via transformation, conjugation, and transduction processes [100,101]. These processes are responsible for the increasing antibiotic resistance worldwide (because of gene transfers between different bacteria species). LGT has been implicated in the distribution of numerous antimicrobial-resistance determinants, and as the cause of an epidemic in nosocomial and community infections, by conferring resistance to many classes of antimicrobials, which leads to multidrug resistance [102,103]. Moreover, the employment of broad-spectrum antibiotics creates selective pressure on the bacterial flora, thus increasing the advent of multidrug-resistant bacteria which results in the production of new antibiotic-resistant bacteria with cycles of unpleasant treatments [70].

(a) Prevalence of antibiotic resistance in some environmental sources

Several authors have investigated the prevalence of antibiotic resistance of some bacteria in different environmental samples. These include the following: Ejo et al. [104] in their findings observed an overall prevalence rate of 5.5% of *Salmonella* isolates identified from raw meat, eggs, milk, and minced meat and burger samples in Ethiopia. The isolates demonstrated relative resistance to ampicillin, tetracycline, and sulphamethoxazole–trimethoprim, with a prevalence of 47.6%. Rasheed et al. [105] equally noted an overall incidence of 14.7% of drug-resistant *E. coli* obtained from vegetable salad, unpasteurized milk, raw chicken, raw meat, and raw egg surface, with 4% of these isolates exhibiting the extended-spectrum β-lactamase activity. In addition, Carballo et al. [106] recovered three tetracycline residues and sixty-three antibiotic-resistant Gram-negative bacteria that presented with percentage resistance between 33.3% and 66.7% to five well-known antibiotics employed in livestock farming, viz. tetracycline, chloramphenicol, nalidixic acid, sulphamethoxazole, and ampicillin. 

Similarly, Zhu and colleagues [107], in their findings, noted the high levels of tetracycline concentration in manure and soil samples procured from three large commercial swine farms, from three different regions in China. The authors further revealed a great diversity of antibiotic resistance genes (149 unique ARGs), and emphasized the absolute abundance of 43% of the aminoglycoside phosphorylation gene *aphA3* in all the manure samples. In the same country, Gao and co-workers [108] equally unravelled the cefotaxime (CTX)-M gene as the most prevalent extended-spectrum beta-lactamase (ESBL) gene found in *E. coli* isolates recovered from both pig farm and soil samples. Apparently, Xiao et al. [21], in a metagenomics analysis of paddy soils from China, provided a broad spectrum profile of antibiotic resistance genes, with multidrug resistance being the most dominant at a level of 38–47.5% of all the samples collected.

Furthermore, Lin et al. [109] isolated and characterized one hundred and thirteen enteric bacteria from the Mhlathuze River, KwaZulu-Natal province, South Africa. Of these bacteria, 75.2% were multidrug resistant, and the enteric isolates obtained from downstream (urban and industrial regions) exhibited greater antibiotic resistance, unlike those from upstream (rural vicinity). This suggests that environmental, industrial, and human activities have a huge impact on the level of environmental antibiotic resistance. Wahome [110] noted the microbial contamination of groundwater samples obtained in Ongata Rongai, Kajiado North County, Kenya, with enteric pathogens including *Pseudomonas aeruginosa*, *Shigella* and *Vibrio* species, *E*. *coli*, and *Salmonella*. The enteric pathogens exhibited high resistance, between 87.5% and 98.5%, to ampicillin, kanamycin, and sulfamethoxazole.

(b) Methods of determining antibiotic resistance of bacterial isolates

The antibiotic resistance profile of bacterial isolates to available antibiotics can be determined by using multiple culture-based methods, with the key feature to isolate the target organism through growth on general multipurpose or selective and/or enriched microbiological media, and subsequent evaluation of their growth in response to specific antibiotic concentrations. The culture-based methods offer a link between antibiotic resistance measurement both in the environment and human clinical setting [111]. These cultured-based techniques are designed as susceptibility tests, and the resistance of the bacterium can be deduced directly from the susceptibility testing. The antibiotic susceptibility test involves both qualitative diffusion and quantitative dilution methods, amongst which is the Kirby–Bauer disc diffusion technique that implements the guidelines adpoted from Clinical Laboratory Standards Institute (CLSI) [112]. In this methodology, the size (diameter) of the zone of inhibition developed around each disc placed on plates of microbiological medium inoculated with the pure culture of bacteria isolate is considered as the degree of sensitivity [113]. The antibiotic susceptibility test with disc diffusion test is, however, regarded as a qualitative test to classify an organism as being susceptible or resistant, and paves the way for the better quantitative tests.

Determining the minimum inhibitory concentration (MIC) of an antibiotic against a bacterial isolate ensures the best quantitative estimate of susceptibility. The MIC describes the lowest or least concentration of a drug that is required to inhibit visible bacterial growth after overnight incubation [114]. The MIC value gives an insight into the degree of resistance, the resistance mechanisms, as well as the resistance genes. It can be determined both by micro broth dilution in microplates [115], and agar dilution [116]. In between, the E-test (Epsilometer test) combines the diffusion and dilution theories in susceptibility testing, whereby it determines whether the isolate is resistant or susceptible, based on the clear zone of inhibition. At the same time, it quantifies the sensitivity of the isolate by giving a discrete MIC value at the point where the clear zone of inhibition intersects on the test strip that harbours a predefined gradient of continuous antibiotic concentration ranges [117]. Nevertheless, owing to the inherent ability of microbiological agars to detect contamination of inoculum, Jenkins and Schuetz [116] suggested that agar-based methods are more reliable for the detection of antibiotic resistance, unlike the broth dilution methods. In general, both the agar-based and broth dilution methods are faced with challenges, including cost, and that they are time-consuming and labour intensive [111]. It is worth mentioning that the resistance level of a bacterium greatly depends on the type of test and test conditions applied for the determination of resistance, as well as the kind of antibiotics and its mode of action [118]. It is necessary to conduct continuous surveillance of the antibiotic resistance profile of bacteria, since bacteria are vulnerable to develop unpredictable resistance patterns, based on their genetic plasticity. In addition, the susceptibility patterns of a particular bacteria changes with time, geographical location, country, and the prevailing environmental conditions [16,119]. The recovered information gives knowledge and serves as guidelines in antibiotic selection for treatment, to reduce the use of broad-spectrum antibiotics, and also to slow down resistance development, hence predict future resistance in bacterial isolates [113,120].

(c) Zoonoses and agriculture versus clinical setting

Taking into consideration the routine use of the same antibiotics with similar modes of action both for animal and human purposes, added to the report of zoonoses, which are bacterial infections in humans caused by animal pathogens, including *Salmonella* spp., *Yersinia enterocolitica*, *Listeria monocytogenes*, *Staphylococcus* spp., *Campylobacter jejuni*, *Enterococcus* spp., and *Escherichia coli*, it is somewhat obvious that antibiotic resistance can be transferred from animals to humans [121]. These bacterial pathogens are of prime importance due to their public health implications, are easily detectable indicator organisms which signify the presence of fecal contamination of the environment, have the potential to acquire resistance genes via lateral gene transfer, and lastly, they can develop resistance to a broad spectrum of last-resort antibiotics [122]. Wegener [123] affirmed that the public health consequences perpetrated by zoonotic pathogens are ever-challenging to evaluate. The consequences involve complex production and distribution systems of food and animals, dissemination of resistance genes and bacterial clones, increased mortality and morbidity, and higher cost in the treatment of disease as well as infections that would have otherwise not have occurred.

These zoonotic pathogens develop resistance in response to the antibiotics used in food animals, and the same strains colonize both animals and humans, and the antibiotic resistance genes can easily spread among the bacterial species or clones that are phylogenetically related [124]. Therefore, these zoonotic bacteria can serve as vectors of antibiotic resistance genes. The resistance can be transferred from animal to animal, or animals to humans, either directly via contact, or indirectly through the food chain, water, sludge-fertilized soils and manure [125]. More specifically, humans can come into contact with antibiotic-resistant bacteria and resistance genes either directly via immediate exposure to animals and biological substances, including urine, feces, milk, semen, and saliva, or indirectly via contact or ingestion of contaminated animal-derived food products [126]. This is, however, the main route for the cause of enteric infections in humans with the zoonotic bacterial pathogens listed above. On the other hand, antibiotic-resistant bacterial strains can be transmitted from humans, including the workers on the farm and their families, to food animals, since it is noted that the digestive tract and the skin of these humans harbour high numbers of commensals, notably *Staphylococcus aureus* [127]. Notwithstanding, the feasibility of transmission is reliant on geographical location, ethnic/cultural practices, religion, hygienic status, farm size, and the type of integrated farming [128]. However, this status quo is true in the rural settings in developing countries, where there is close contact between the animals (specifically, poultry) and humans. Since farming of indigenous-species animals in small numbers appears to be the most common practice of poultry production systems, in this regard, the animals depend on scavenging as a source of food [37,129]. Also, in the developing world, biosecurity and food safety measures are inadequate, thus facilitating the direct and indirect mode of acquiring antibiotic-resistant bacterial strains and their resistance genes. Kagambéga et al. [130] isolated and characterized *S*. *typhimurium* from poultry and humans that were resistant to the same antibiotics, and harboured the same phage, DT 56, which demonstrated close-relatedness as revealed by pulse field gel electrophoresis.

Lipsitch et al. [131] presented three mechanisms through which antibiotic resistance originating from agriculture can threaten human health, as follows: an individual might be infected by a resistant bacterial pathogen via direct contact or via ingestion of contaminated meat, milk, eggs, or water, and not transmit to other humans. In another scenario, a person might be infected with a resistant pathogen via the aforementioned pathways, with ongoing transmission to other humans and causing infections in some of the individuals. Thirdly, resistance genes derived from the agricultural settings are being introduced into human pathogens by lateral gene transfer. Apart from acquiring resistance by these bacteria, they can equally receive additional virulent genetic elements, leading to an increase in pathogenicity or virulence [39,132].

Wegener et al. [133] emphasized that the observed level of antibiotic resistance is closely associated with the amount that is being consumed. Apparently, the level of antibiotic resistance of these bacteria relies on the quantity and the effects of antibiotics in the natural environment originating from antibiotic management within the agricultural and healthcare settings, as well as the antibiotic prescription guidelines. According to Sahoo et al. [16], the antibiotic prescription is influenced by geographical and climatic factors, socioeconomic conditions, local population density, the behaviour of a particular community towards antibiotic prescription or consumption, and supplier incentives to the prescribers in conjunction to the type of pathogen.

All the same, antibiotic resistance has adverse effects on patients, healthcare systems, and society [134]. More specifically, patients witness a more severe underlying infection, are administered less efficacious but more toxic antibacterial agents, and receive broad-spectrum antibacterial agents, which are so-called reserved or last resorts as an empirical antibiotic regimen [132]. These might result in treatment failure, as well as an increase in the cost of human therapies, due to the severity and persistence of the diseases, added to long hospital stay and prolonged therapy, respectively [135,136]. Antibiotic resistance equally limits the choice of antibiotics to be implemented in therapy and jeopardizes the chances of the effectiveness of the existing potent antibiotics in treatment regimens used for the eradication of serious but common diseases in the future [137]. The rising level of antibiotic-resistant bacterial pathogens will eventually hamper future treatment and the prevention of infectious diseases in both animals and humans [138]. Also, the incidence of antibiotic resistance is very critical to the immunocompromised population, since these individuals rely solely on the use of antimicrobials as a defense against pathogens [139]. Unfavourably, the rise in antibiotic resistance presents a potential threat to surgical and advanced therapeutic procedures, including transplantation or anticancer therapy that involve immunosuppression. Therefore, these medical procedures need vigorous anti-infective preventive therapies [2,134]. However, antibiotic resistance can cause the death of individuals which is the most severe outcome [134].

(d) Principles of antibiotic use and antibiotic resistance in the clinical and agricultural sectors in both developing and developed countries

The principles or standards established to guide antibiotic use in both agricultural and clinical settings vary between the developed and developing countries, as well as differs from one country to the other. This is visualised from the variation in the antibiotic consumption pattern across the globe, highlighted by Van Boeckel et al. [12]. It is greatly influenced by the antibiotic policies which govern antibiotic use concerning the antibiotic manufacture, antibiotic dispensation, and antibiotic prescription (inappropriate choice and dosing of drugs) of a particular country [140]. However, antibiotic policies are negatively affected by the socioeconomic level (infectious disease burden, income level, educational status, etc.), large population size, and heterogeneity disparity in the healthcare systems in developing countries [11].

The dearth of functioning antibiotic policies has culminated in the inappropriate use of antibiotics. Van Boeckel et al. [12] stated that about 50% of antimicrobials are used incongruously, regardless of the setting owing to lack of antimicrobial stewardship. Antimicrobial stewardship (formerly called antibiotic policy) entails the choice, dosing, route, and duration of administration of a particular antimicrobial agent. It is defined as the administration of the right drug at the right dose, through the right route, at the right time, to the right patient, to ensure the best clinical outcome for treatment or prevention, thereby causing least harm or toxicity in the patient and future patients [141]. The leading goal of antimicrobial stewardship is to optimize clinical outcomes, to maximize clinical cure or prevention, and to limit the unintended penalties of antibiotic use, including toxicity and the emergence of antibiotic-resistant bacteria [141,142].

From the clinical perspective, antibiotics are the most widely used therapeutic agents worldwide. To avoid the irrational and unnecessary use of antibiotics, it is imperative that antimicrobial stewardship is implemented regarding prescriptions guided by appropriate principles based on the patient’s characteristics, the characteristics of the disease-causing agent, and the colonizing microflora [142]. More specifically, that the pharmacokinetics and pharmacodynamics of the drug, as well as host factors are not left out, in an appropriate antibiotic therapy. The microbiological diagnosis presents as the key procedure in any therapeutic process, where the etiologic agent and the antimicrobial susceptibility patterns, as well as surveillance of the resistance of the pathogen are conducted. Appropriate specimens are collected and submitted to the microbiology laboratory for diagnosis. Diagnosis, in most cases, often relies on culture-based methods which are time-consuming and take several days for a positive result to be obtained. As a consequence, other rapid microbiological methods, including rapid polymerase chain reaction and mass spectrometry have been adopted, and stand a better chance for the future [143].

In addition, new and rapid molecular tests, including peptide nucleic acid and matrix-assisted laser desorption/ionization technologies have been introduced, that identify common organisms from positive cultures within several minutes [141]. However, especially in critically ill patients (e.g., endocarditis, bacterial meningitis) that are hospitalised, an empirical therapy is decided guided by the clinical presentation of the patients and the site of the infection, in order to reduce the morbidity and mortality rate. Broad-spectrum antibiotics are used that cover a broad spectrum of suspected and non-susceptible pathogens responsible for the clinical presentation [144]. Usually, the combination therapy relies on the synergistic action of the recommended drugs to clear, more rapidly, the infecting microorganism or infection caused by resistant bacteria to multiple antibiotics, or infections caused by more than one organism, as well as to truncate antibiotic therapy [143]. On the other hand, a definitive therapy is employed following the release of the laboratory results on the specific etiologic agent, with the critical opinion to narrow the antibiotic spectrum. Disapprovingly, the definitive therapy is described as the most important component of the antibiotic therapy, as it optimises treatment, reduces costs and toxicity, added to its great possibility of preventing the development of antibiotic resistance [145]. Also, very important at this junction, the clinician consults the modes of action of these drugs, whether they are bacteriostatic or bactericidal, but in more serious infections, bactericidal antibiotics are preferred. In summary, antibiotics maybe consumed in therapies/treatments as first, second, and third line antibiotics. First line drugs are administered to patients guided by the clinical presentation and antibiotic susceptibility results, and are based on their broad availability, relatively low cost, and tolerance. However, if a patient fails to respond to the initial drugs or develops intolerance to drugs and/or relapse of infection occurs, other drugs, known as the second line, are added to the treatment. Also, if resistance to the second line drugs is observed, third line drugs are included in the treatment, even though these drugs are associated with higher risk of toxicity and other side effects, unlike the first and second line drugs [144].

As indicated above, the use of antibiotics in agricultural settings is not only for therapeutic purposes. The employment of antibiotics in different applications in food animals is described as therapeutic use, prophylactic use, and sub-therapeutic use [146]. Ideally, the use of antibiotics in food animals for therapeutic purposes should be accompanied by antibiotic susceptibility testing (AST). Altogether, the results, the age and immune status of the animal, attributes of the drug (pharmacokinetics and pharmacodynamics), and the cost of the drug, are considered in order to decide on the appropriate drug to be used. As in human medicine, the antibiotics during therapy can be categorized into first, second, and third line drugs [147]. However, with respect to the different animal type and practice, the antibiotics vary in the classification, but the principle still remains that the first line drugs are often used in the treatment of most bacterial infections, and the second and third line options are rarely needed.

However, there is an obvious link between antibiotic use and resistance, both on an individual and population level. The high disease burden, poor hygienic and sanitation conditions, limited access to available antibiotics (due to poverty), disparity in healthcare systems and personnels, over-the-counter purchase of drugs, lack of stringent antibiotic policies (that affect the quality and potency of drugs produced), unregulated prescription principles (that lead to self-mediation and prescription by untrained persons), patient expectations, financial incentives to healthcare providers to prescribe antibiotics in developing countries, cause inappropriate use of antibiotics, resulting in antibiotic resistance [140,148,149]. Thus, there exists differences in the antibiotic resistance levels between and within countries. For instance, Gebeyehu et al. [150] gave an insight into the inappropriate use of antibiotics in the rural (29.2%) and urban (31.1%) communities in North West Ethiopia, and attributed the practice to younger age, involvement with a job, paucity of knowledge on the use of antibiotic preparations of humans to animals, and dissatisfaction with the healthcare services.

(e) Containment of antibiotic resistance or strategies implemented to maintain appropriate use of antibiotics

Nevertheless, across the globe, countries, states, and regions within the countries have implemented several procedures to regulate and reinforce rational and prudent use of antibiotics in both the clinical and agricultural sectors, in order to contain antibiotic resistance. All these procedures are necessary to conserve the available antibiotics and to maintain their effectiveness. Antimicrobial stewardship occupies a central role in the endeavour to avoid misuse, overuse, or abuse of antibiotics in both settings.

I. Clinical sector

In the clinical sector, the front-end or preprescription, and the back-end or postprescription approach are implemented with different techniques/strategies to optimize the use of antibiotics. These techniques include formulary restrictions, order sets and treatment algorithms, clinical guidelines, education, pharmacodynamic dose optimization, computer-assisted decision support system, pharmacist-driven intravenous to oral switch programs, pharmacy dosing programs, and antibiotic cycling [141]. The back-end approach offers as a better option because it uses prospective review and feedback, and focuses on de-escalation, which permits the modification (a change, adjustment/reduction, discontinuation) of initial empirical antibiotic therapy relying on the culture data, clinical status of the patient, as well as the other laboratory results [151]. The antibiotic de-escalation therapy is the key component within antimicrobial stewardship [152].

Apparently, McKenzie et al. [153] demonstrated that antibiotic restriction, education of prescribers and patients, and prescription feedbacks as antimicrobial stewardship strategies have improved with the prudent use of antibiotics in Australian hospitals. In addition, the California state in the United States has instituted antimicrobial stewardship in its state legislation [154]. In addition, some countries (e.g., Brazil and Mexico) have implemented policies to regulate and prohibit the sales of over-the-counter antibiotics without prescription [155]. Also, the Central Drugs Standard Control Organization (CDSCO) in India instigated Schedule H1, a stricter regulation, unlike Schedule 1, in a bid to prohibit the sales of over-the- counter antibiotics. According to Laxminarayan and Chaudhury [156], Schedule 1 harbours antibiotics that must be sold with a valid prescription issued by a registered medical practitioner, and the pharmacist is required to retain a separate register that carries the contact details of the prescribing doctor, the name of the patient, as well as the name and quantity of the drug that is dispensed. The register is kept for three years, and the information contained therein is subject to audit by the government.

Furthermore, the Medicine Control Council (MCC) as part of the National Drug Policy in South Africa subscribes to the World Health Organization Certification scheme. It is mandated to register and relicense, conduct dossier-based medicine evaluation and laboratory-based testing of all medicines utilized in the country in conformity to the criteria for medical evaluation and good manufacturing practice. Moreover, Essential Drug Lists (EDLs) and Standard Treatment Guidelines (STGs) are developed as part of the strategy of National Health policy (NHP) in South Africa, so as to ensure that drugs are readily available and accessible at primary care and at the hospital level, in addition to limiting the choice of antibiotics use via replacement with formularies in the public sector. Thus, the practice results rational prescribing [157]. Gelband and Duse [158] highlighted that as a regulatory strategy in South Africa, only licensed practitioners might prescribe and/or dispense antibiotics, and the antibiotics are available only on prescription, but not bought over the counter like in other developing countries. In the same way, antibiotic use was substantially decreased at the primary care in Thailand, as well as nationwide actions were demonstrated to address the problem of inappropriate antibiotic use through strengthening of hospital drug and therapeutics committee, engagement in a project based on multifaceted behavioural change intervention, and updating of its essential medicine lists on a regular basis [159].

Surveillance is the pivot in any control strategy directed against infections in a clinical setting and antimicrobial resistance. Antibiotic surveillance is regarded as the keystone in endorsing antibiotic stewardship, and eases the control of antibiotic resistance. It is also considered as the force behind the programmes geared towards antimicrobial resistance, since it generates reliable and crucial data that can be used to formulate policies on antibiotic use to promote accurate prescriptions of drugs [160]. According to the Global Antibiotic Resistance Partnership—India [161], real changes in antibiotic consumption or dissemination of resistant bacteria can only be appreciated and/or supported when the resistance level is known and tracked over time, unlike undergoing any type of surveillance. Hence, surveillance of antibiotic resistance complements the surveillance of antibiotic use, and obtained data can be implemented to evaluate the success of intervention programmes. Therefore, salient data required for clinical decision making and national policies can be assembled via surveillance of antibiotic use and antibiotic resistance [162]. 

According to Lowmann [163], the clinical microbiology laboratory undertakes a central role in achieving the key motives of antibiotic stewardship by providing data on culture and susceptibility of the specific patient, and insights for surveillance activities that guides the selection of antibiotics for empirical therapy. Monitoring the consumption of antibiotics is inevitable, as it generates data that can assist in the design and evaluation of interventions aimed at optimizing the use of these antibiotics and prevent rising resistance [155]. The World Health Organisation [164] and the Global Action Plan [165] advocated that the quantity and pattern of antibiotics consumed should be monitored as part of surveillance. Pereko and colleagues [160] analyzed prescription claims data and sales data from 2008 to 2011 in the private sector in Namibia to obtain the number of prescriptions that contained antibiotics and the volume of units sold. The findings highlighted the highest antibiotic consumption by females (53%), followed by individuals of age, 18–45 years (41%) and 34% in Windhoek, with combined therapy of amoxicillin/clavulanic acid as the post prevalent agents used which belong to the family of penicillins.

On the other hand, surveillance of antimicrobial resistance is equally paramount, and should be conducted continuously in order to gain insight into the problem on time, because resistance is evolving. It is defined as the continuous, systematic gathering, analysis, and interpretation of health-related data to monitor and describe an event [166]. Surveillance provides current and salient information needed to develop and monitor antibiotic stewardship programmes, antibiotic formularies, infection control policies, public health interventions, novel antimicrobials, and antibiotic therapy guidelines [167]. It can be executed at the local, regional, national or international level, involving the monitoring of a single bacterial infection or the organism, as well as it can be effected based on funds offered by companies or non-profitable organizations [168,169]. The procedure provides data on the antibiotic-resistant bacteria, resistance genes, and predict the rise of antibiotic resistance as depicted from the trend in the antibiotic resistance profiles. Surveillance represents an early-warning system which performs fast distribution of crucial information to public health and regulatory authorities regarding trends and possibility of outbreaks, to bring about timely response measures [170]. Also, Essack [168] reiterated that antibiotic resistance surveillance data are critically needed to update national infection control policies, essential drug lists, and standard treatment guidelines. In addition, Critchley and Karlowsky [170] further emphasized that the data obtained from surveillance and clinical trial can be employed in association to determine breakpoints. Moreover, new medical needs, as well as notorious isolates for the screening of new agents, can be identified from the information contained in surveillance of antibiotic resistance.

In Africa, South Africa is viewed as the only country with the most active surveillance system. The system comprises of two main active groups, namely; the Group for Enteric, Respiratory and Meningeal Disease Surveillance (GERMS) which focuses on obtaining data centered on AIDS-related opportunistic infections, epidemic-prone diseases, and vaccine-preventable diseases. On the other hand, the South African Society for Clinical Microbiology (SASCM) assembles data from the academic hospital on preferred invasive pathogens secluded from the cerebrospinal fluid samples [171]. Aside from these two groups, extra contributions in the collection of data on antibiotic resistance are given by other groups, including the Sexually Transmitted Infection (STI) Reference Centre in alliance with the National Department of Health, the Enteric Diseases Reference United (EDRU) etc. during surveys. It is worth mentioning that the Gonococcal Antimicrobial Surveillance Programme (GASP) co-ordinated by WHO in Africa was accomplished, owing to the principal role performed by the STI Reference Centre. The STI Reference Centre has provided training and technical assistance in isolate collection and laboratories in Madagascar, Tanzania, Zimbabwe, and Namibia [158].

Furthermore, in Nigeria, the surveillance system for antibiotic resistance in pulmonary tuberculosis referred to as the National Tuberculosis and Leprosy Control Programme (NTBLCP) is commended by Nasir and co-authors [169] as the only functioning system in which services for drug-resistant TB is provided by four reference laboratories of the country. Also, WHO [172] has introduced the nested PCR equipment in numerous healthcare facilities in Nigeria, therefore, facilitating the molecular-based anti-TB resistance testing regarding rifampicin resistance. Xiao and Li [173] also noted that the Chinese government embarked on a three-year special campaign with the theme “Administrative regulations for the clinical use of antibiotics” to enhance the rational use of antibiotics in 2011. The outcomes of a survey conducted at the tertiary hospitals from 2010 to 2012 involved reductions in antibiotic consumption during prophylaxis in surgical processes (95% to 44.6%), as well as in antibiotic prescriptions to outpatients (22% to 14.7%) and inpatients (68.9% to 54%).

II. Agricultural sector

Antibiotic use for essentially non-medical or non-therapeutic purposes in agricultural settings that are at subtherapeutic levels over an extended period is observed as a major route for the advent of antibiotic resistance and antibiotic-resistant bacteria, and resistance genes have been reported to be transferred to humans [174]. Irrational or non-prudent use of antibiotics in food-producing animals have resulted in antibiotic residues in animal-derived products. Therefore, antimicrobial stewardship is equally implemented to ensure prudent antibiotic use in agriculture, in order to conserve and maintain the effectiveness of available antibiotics, as well as curb the problem of antibiotic resistance and residues in food products derived from animal [175]. Sadly, stewardship interventions in developing countries have often been weakened by the attitude of poorly paid veterinarians who seek supplementary incomes from drug sales, plus the existence of inadequate regulations [25,176]. Summarily, all stakeholders involved in the fight against antibiotic resistance must address it from the stand point of regulations, surveillance, research, treatment guidelines, infection control, education, and awareness [177]. The National Farmed Animal Health and Welfare Council [178] pointed out that the implementation of antimicrobial stewardship in agriculture can be approached from the following perspectives, including clinical microbiology, infection control (biosecurity), regulations, surveillance on antibiotic use and resistance, animal management, husbandry, and alternatives to antibiotics. A coordinated network of actions from the veterinarians, livestock producers, pharmacists, veterinary pharmaceutical industries, and regulatory authorities are relevant to enforce prudent antibiotic use [179].

Following the Global Action Plan on antimicrobial resistance [28] and the Global principles for the containment of antibiotic resistance in animals intended for food presented by WHO [180], nations are expected to implement measures that are in line with the key actions highlighted for the combat of antimicrobial resistance. Accordingly, Walsh and Wu [181] expressed the interdict on the use of colistin (an antibiotic critical for the treatment of infections caused by highly resistant Gram-negative bacteria in humans) as a feed additive for animals in China. More elaborately, some European countries, including Sweden, Denmark, United Kingdom, Netherlands, etc. introduced bans on the use of antimicrobial growth promoters [36], while Australia and New Zealand implemented a partial ban. Seemingly, in the United States, the restraint on antimicrobial growth promoters is voluntary. In addition, a tripartite alliance involving WHO, World Organisation of Animal Health (OIE), and Food and Agricultural Organisation of the United Nations (FAO) was formed in 2003, which led to the categorization of veterinary medicines into critically important, highly important, and important drugs for human health. This differentiation is to guide their use in animal agriculture across the globe, hence, combating antimicrobial resistance. 

Also, some developing countries do not have national limits for antibiotic residues found in animal-derived food, and thus, rely on international maximum residue limits (MRLs). Therefore, in this continuous fight against antibiotic resistance, the joint FAO/WHO expert committee and Codex Alimentarius Commission updated the MRLs of veterinary drugs in foods of animal origin [79]. Likewise, the Ministry of Health and Family Welfare in India amended the Drugs and Cosmetics regulations, 1945, and set withdrawal limits for drugs used in animal farming. The ministry further emphasized that drug producing companies should equally imprint the withdrawal periods on the containers of drugs meant for animal consumption; however, if not provided, a withdrawal period of not less than 28 days should be considered [182].

It is worth mentioning that the drug delivery system and the priorities and demands of a country vary from one country to another across the world, thereby causing governments to outline regulations or policies or measures according to their local scheme [183]. Mehrotra et al. [184] found that Health Canada has strengthened the veterinary drug regulatory framework in a bid to enhance antibiotic stewardship. These were accomplished via increased control over imported veterinary drugs and active pharmaceutical ingredients (API), a compulsory annual compilation of veterinary antibiotic sales by manufacturers, importers, and compounders. Lastly, it created easy access to low-risk veterinary health products (e.g., vitamins, botanicals, traditional medicines, minerals) which serve as alternatives to antibiotics.

Also, antibiotic prescription and administration to farm animals are supervised by veterinarians, and several behavioral studies have proven that the attitude of farmers to antibiotic use is greatly influenced by the veterinarians. Therefore, interventions geared toward the change of prescribing behaviour of the veterinary could go a long way to optimize antibiotic use by farmers. However, the determinant factors, including a personal opinion regarding the contribution of veterinary medicines in antibiotic resistance, professional ethics to alleviate animal suffering, financial dependence on clients, amongst others, have been noted to influence the prescribing behaviour of the veterinarian [185]. Also, Henton et al. [186] emphasized that the registration of over-the-counter drugs for sales is not optional in South Africa, and the drugs are distributed by the manufacturers to veterinary wholesalers, farmers’ cooperatives, distributors, and feed mix companies. Also, Lee et al. [187] affirmed that educational programs should be conducted for undergraduate medical and non-medical students in line with generic medicines, mechanism of antibiotic resistance, and prudent use of antibiotics.

Strategies to reduce or limit the therapeutic use of antibiotics in animals via improved animal nutrition, improved living conditions and waste management, biosecurity measures, and improvement in animals’ natural immunity can result in infection prevention and control. These strategies, however, will reduce the level and type of antibiotic needed for treatment, because once the animal is exposed to infection, its immune system can fight seriously against the agent, resulting in less severe manifestations [176]. Moreover, reductions in antibiotic consumption can be achieved by using non-antibiotic alternatives, including prebiotics, probiotics, bacteriocins, vaccines, innate immune system potentiators, bacteriophages, and competitive exclusion cultures for non-specific and specific control of enteric pathogens in animals [188]. Nevertheless, guided interventions, such as vaccination, antihelminthic therapy, optimized herd management, improved biosecurity measures, prudent antibiotic use, performed as teamwork involving the farmers and veterinarians, have led to a marked curtail in antibiotic use, especially the critically important antibiotics, with a decrease of 52% and 32% of the pigs from birth to slaughter and breeding animals, respectively [189].

According to Silbergeld et al. [190], the largest consumption of antibiotics is found in the production of food animals. The documentation of resistant and multidrug-resistant pathogens in food animals poses threats to public health and food security [191]. Also, the findings of the investigation conducted by Chantziaras et al. [192] revealed a strong correlation between the level of specific antibiotics used and the level of resistance in *E. coli* strains recovered from pigs, cattle, and poultry of seven European countries. Therefore, data collection of antibiotic use is necessary to understand and counteract antibiotic resistance. The monitoring of antibiotic use is crucial in determining the appropriateness of the drugs in use, detect compliance with prudent drug practice, programmes, or regulations [193]. It can equally contribute in the process of optimizing antibiotic use by identifying the most efficient interventions. In addition, monitoring of antibiotic use creates tolerance in analyzing the temporal trend of antibiotic consumption, and of remarkable interest is the data collected on antibiotic use per animal species. For instance, Schaekel et al. [194] investigated the distribution of antibiotic use per pig age group and active substances (in percentages) used in overall treatment in pig holdings in Germany over a two-year period. The authors found that the types or combination of antibiotics employed varied between the age groups, and the fattening pig experienced a decreasing trend in treatment, but no distinct temporal trend was obtained for the weaners and sucklers. The quantification of antibiotic use in conjunction with data on antibiotic resistance can pave the way for targeted research and development. This is because the data can be used to describe temporal associations between antibiotic use and antibiotic resistance, as well as offer evidence for their links to researchers and policy/decision makers [195].

Surveillance produces reliable data, and it can be approached from various angles; the antibiotic resistance of pathogens or commensal bacteria, antibiotic use in the treatment of diseases and non-therapeutic uses [196]. The surveillance systems for antibiotic consumption are not standardized and differ from one country to the next. These systems may include; DANMAP (Danish Integrated Antimicrobial Monitoring and Resistance programme, Kongens Lyngby, Denmark), MARAN (Monitoring of Antimicrobial Resistance and Antibiotic usage in Animals in Wageningen, The Netherlands), and SANVAD (South African National Veterinary Surveillance and Monitoring Programme for Resistance to Antimicrobial Drugs, Pretoria, South Africa). Systems designed for antibiotic consumption can be automated systems or randomized field studies, as well as nationwide or cross-sectional or longitudinal studies, which focus on analyzing the sample population of farms [194]. On the other hand, the cross-sectional studies can be differentiated into national or regional studies. Overall, these studies are heterogenous, and cannot be compared as they consist of differences, including the duration of data collection (yearly, half a year, or even days), different calculation and reporting methods, along with the stratification in the production systems and farm types [194]. Furthermore, some systems monitor and collect data only at the farm level, making it possible for the implementation of benchmarking approaches, whereby farmers or veterinarians are compared and ranked as stated by their level of antibiotic use. By so doing, measures that are relevant to reduce consumption in the top antibiotic users are introduced or implemented [197]. 

In addition, Adesokan et al. [198] surveyed the different classes of antimicrobials administered to food animals in South Western Nigeria, and noted that tetracyclines, fluoroquinolones, and followed by beta-lactam/aminoglycosides, were consumed in order of decreasing volume, and mostly in rainy season than dry season. In addition, World Organisation for Animal Health highlighted that 94% of African Countries lack an official surveillance system on the use of antimicrobials in animals [199]. However, though the survey of antimicrobial use in developing countries is inadequate, it demonstrates a high level of farmer prescription, purchase of over-the-counter antibiotics, non-compliance to antibiotic withdrawal periods, and drug counterfeiting [25,167,175].

Surveillance of antibiotic resistance is a vital tool for microbial risk assessment, and the data generated enlighten the understanding of resistance epidemiology, as well as calls for prompt, effective, and early control actions from the government, and also monitor and evaluate the effectiveness of policies implemented by the government [200]. According to Dar et al. [201], and Gelband and Duse [158], only a few national, cross-sectional studies on antibiotic resistance of animal-recovered isolates have been carried out in low- and middle-income countries and South Africa, respectively. However, the surveillance systems differ between countries and/or agencies, due to variation in agricultural practices, monitoring needs, and available guidelines [177]. Furthermore, an integrated surveillance of drug-resistant bacteria obtained from humans, food-producing animals, food and other environmental sites, synchronized with the monitoring system for antibiotic use, can assist in the containment of antibiotic resistance. This is described as the “One Health Approach” intended to attain optimal health for humans, domesticated animals, wildlife, plants, and our environment [29,196]. The first ever integrated animal–human surveillance system was created in Denmark, known as DANMAP (Danish Integrated Antimicrobial Monitoring and Resistance Programme) which addresses the problem of antibiotic resistance with bacteria of food, animal, and human origin [201].

III. Challenges Faced by Developing Countries Regarding Surveillance Systems

A satisfactory and comprehensive survey is needed to understand the epidemiology of antibiotic resistance in both animals and humans. Even though the global action plan by WHO/OIE/FAO to contain antimicrobial resistance requires each nation or country to implement national action plans, developing countries are still to develop a sustainable surveillance system of antibiotic use and antibiotic resistance [202]. The challenges regarding the surveillance system include:

1. The countries have just a few laboratories with the potential to conduct quality-assured microbiology and drug sensitivity testing. Ǻrdal et al. [203] strongly affirmed that collection and reporting of data and the strengthening of laboratory capacity are the two related issues in surveillance.
2. Due to the high burden of infectious diseases and low socioeconomic status of these countries, there is a lack of available resources [204]. According to Nasir et al. [169], the developing countries lack the funds to purchase reagents and consumables essential for testing antibiotic resistance, thus lack necessary plans for the surveillance of antibiotic-resistant bacteria. The cost necessary for the adequate surveillance, together with the small margin profits in the veterinary sector presents a financial drawback to support surveillance in the veterinary and agricultural sector [166].
3. There is a discrepancy in the selection of isolates. Most of the isolates are from clinical cases that are sick individuals (human or animals). Therefore, the sample of isolates is biased toward a more resistant isolate, owing to the previous antibiotic therapy administered. Also, only a few isolates are involved, since the veterinarian decides on the individual animals to refer to the laboratory. Consequently, the proportion of the isolates is not a representative of the bacteria strains under survey taken from animals [200].
4. Also, relatively few studies have been conducted on animal-recovered isolates, as well as the criteria for testing isolates differ between countries, likewise, the antibiotics that are tested [158,201].
5. Either at the regional, national, and local levels, there exists variation in obtaining data, owing to differences in laboratory protocols, conditions employed for testing, personnel conducting the drug sensitivity assay, antibiotic policies, quality control and assurance of the laboratory, and considerations regarding breakpoints. In reality, there are no well-known breakpoints for the animal, which results in the adoption of breakpoints values from human medicines. Nevertheless, the standard protocols and breakpoints from the Clinical Laboratory Standard Institute (CLSI) have been adopted by most countries [200].
6. These countries also lack stringent and comprehensive policies and plans to circumvent antibiotic resistance. They lack enforcement of regulations regarding prudent antibiotic use, since many are still faced with the problem of purchasing drugs over the counter or without a prescription [161], and the presence of counterfeit drugs [158,196]. Nevertheless, even if some data are collected, they fail to translate the surveillance data into policy, especially in South Africa [9].
7. At the national level, there is a lack of collaborative measures between the different laboratories regarding surveillance of antibiotic resistance, which might hamper efforts to track emerging resistance and also limit the chances of systematic comparison and evaluation of national activities directed toward the containment of antibiotic resistance.

### 3.1. Antibiotic Resistance in Livestock Farming 

According to Woolhouse et al. [205], antibiotic resistance in livestock farming can be looked at from four different viewpoints, i.e., the animals (cattle, pigs, poultry, sheep) and animal-derived products, farm workers, and farm environmental sites (water, soil, feeds, wastewater, sewage, lagoon, manure, and sludge after treatment). All these constitute the several compartments and different niches in the farm described as an ecosystem [127]. Undoubtedly, farm animals are a very important component in understanding the interplay between humans, animals, and the environment regarding bacteria, antibiotics, and antibiotic resistance gene movement [205]. The digestive tract of animals, like humans (farm workers), is colonized with diverse microorganisms, including commensals and resistant bacteria. Thus, it serves as the most important reservoir of microorganisms. Therefore, it can play a vital role in the dissemination and acquisition of resistant bacteria and their resistance genes [127]. 

Shobrak and Abo-Amer [206] noted the occurrence of multidrug-resistant *E*. *coli* and *E. vulneris* in cloacal samples of both migrating and non-migrating birds which served as carriers. Through the release of their fecal residues into water bodies and other environmental sources, it could enable the spread of these resistant bacterial strains and their resistance determinants even to remote areas by means of migration. The anatomical feature of the gut varies between poultry and the other mammalian animals, which in turn influences the intestinal microbiome [207]. The intestinal microbiome changes with age, the type of diet fed, antibiotics ingested, infection with pathogens, amongst other life events [208]. It has been reported that microorganisms in the gut interact extensively with the host, diet, and the intestinal gut microbes, and exert a huge impact on the animal’s immunity and physiology, and ultimately affects the health of the animal and its production [209]. 

The continuous antibiotic exposure to animals via oral administration creates selective pressure for the development of resistance, and resistant bacteria associated with animals can then enter into the food chain through the consumption of meat (contaminated during slaughter or processing of carcass, if the gut is accidentally cut or intestine empties its contents into the thoracic and abdominal cavities when the carcass of poultry is gutted during processing) or other animal-derived products, through farm runoff water and other means. However, the greater the quantity of antibiotic used, the higher the selective pressure. Van et al. [210] reiterated that food contaminated with antibiotic-resistant bacteria could cause amplification of resistance genes and facilitate the transfer of the antibiotic resistance determinants to other bacteria of clinical importance found in humans, and can be further transferred within humans to more pathogenic bacteria. Therefore, food or animal-derived products, including meat, milk, and eggs, may represent an active and key medium through which antibiotic resistance determinants are continually being transferred between bacteria, and from animals to humans [123]. Bosco et al. [211] clearly documented the multidrug resistance of *Salmonella* isolates recovered from cattle, pigs, chickens, eggs, and animal-derived products, as well as cross-species transmission of plasmids between animal and humans in Uganda.

Also, the farm environment is composed of environmental sites, such as manure, wastewater, soils, effluent, and sewage, which serve as hotspots for antibiotic resistance pollution. More specifically, Bester and Essack [39] indicated that animals are very exposed at a high degree to their environment, making it easier for them to be infected with bacteria harboring problematic genetic material, especially from the soil environment. In the same light, animal urine and feces containing antibiotic residues, antibiotic-resistant bacteria, and resistance genes may be released into manure, and animals might, in turn, be allowed to graze on pasture grown on soil fertilized with this raw manure. This, therefore, creates the likelihood of bringing back these xenobiotics to animals and humans [127]. 

Nevertheless, manure, which has been described as a hotspot for antibiotic resistance bacteria and antibiotic resistance genes, can serve as a plausible route of transmission of these resistant bacteria and their genes into the soil and water via deliberate or accidental processes [212]. Therefore, it is advised that manure should be treated before land application via biological methods, including anaerobic digestion. However, Resende et al. [213] in their findings noted the prevalence and persistence of potentially pathogenic bacteria which demonstrated multidrug resistance against oxacillin, ampicillin, and levofloxacin, amongst other antibiotics, both in the influent (cattle manure) and effluent (digestate) released from an anaerobic biodigester. The authors suggested that the rate of survival of these bacteria depended on the temperature of the operating process in association with the duration of the fermentation process and the microbial composition. Ostensibly, Maynaud et al. [214] further confirmed the occurrence of enteric pathogenic bacteria in the digestate obtained from the anaerobic biodigester. The authors emphasized the potential of the viable, but non-cultural state of bacteria, which might cause the regrowth of pathogens during digestate storage, prior to land spreading. Consequently, the need of post treatment of digestate via mechanical, chemical, physical, and biological methods is very vital [215]. In addition, the sanitary risk and microbiological safety of digestate should be evaluated before land application of digestate, in a bid to dodge the ecological, human, animal, and environmental health implications. Nevertheless, it is a call for concern when pathogenic bacteria are present alongside with antibiotic-resistant bacteria in untreated or treated animal manure.

Alternatively, the very common and economic approach to manage manure generated from livestock farming is by application on nearby agricultural fields. However, raw manure can be flushed by heavy rainfall or runoffs from the surface of manure-amended soils into nearby water bodies used by humans for sanitation and domestic purposes [216]. Moreover, due to the constraints on available freshwater resources in developing countries, wastewater serves as a vital source of water and nutrients for irrigation of agricultural fields, in a bid to circumvent the problem of food insecurity in these countries [217]. However, wastewater may infiltrate into groundwater, causing pollution and contamination with toxic chemicals, antibiotics, and organic matter. In addition, owing to the lack of or inefficient wastewater treatment plants, wastewater or improperly treated wastewater is mostly released into surface water bodies that act as reservoirs for domestic and industrial wastes, causing pollution [218].

### 3.2. Antibiotic Resistance in the Soil Environment

The soil is an ecosystem and a natural resource with unique biodiversity, taking into consideration abundance, quantities of species, and functions of organisms [218,219]. With respect to total biomass, microorganisms are considered as the principal part of the soil community, and are basically responsible for decomposition of organic matter, degradation of toxic compounds, and nutrient transformation [218]. Interestingly, the soil is composed of microorganisms that produce antibiotic by so doing; it can equally serve as a reservoir of antibiotic-resistant bacteria and resistance genes [220,221]. The soil serves as a hub to establish connections between the air, water, rocks, and organisms, and it is involved in many different functions termed ecosystem services in the natural world [222]. Thus, it can be described as quite a large reservoir of antibiotic resistance determinants, since it includes the antibiotic resistance determinants found in all plants, fungi, soil bacteria, small animals, and protists [222]. In addition, Reisenfeld et al. [20] indicated that uncultured soil bacteria are a possible reservoir of antibiotic resistance genes with greater diversity as compared to previous findings, and such diversity can be ascertained and fathomed using culture-independent methods. Furthermore, vegetables grown in unfertilized soil were equally shown to harbour antibiotic-resistant bacteria and resistance determinants that naturally occur in soils [97].

It is somewhat obvious that the abundance and the mobility of antibiotic-resistant bacteria and resistance determinants in the soil can be greatly influenced by the application of manure (containing antibiotic residues, antibiotic-resistant bacteria, and their resistance genes on mobile elements) during fertilization of the soil, the use of wastewater (black or grey water) for the irrigation of agricultural lands, and the use of antibiotics to treat crop diseases [4,223]. More specifically, when soils are treated or amended, antibiotics and their degraded metabolites, as well as antibiotic-resistant bacteria and their corresponding resistance genes, are introduced into the soil environment. Therefore, transfer of antibiotic resistance genes becomes inevitably fast, owing to the rapid growth of bacteria and HGT [224]. However, the persistence and the rate of dissipation of the antibiotic resistance genes is dependent on the VGT/HGT of ARG, the transport and viability of the bacteria harbouring the genes, whether the free DNA obtained from cell lysis will be degraded, adsorbed to soil or organic matter, or acquired by new cells, as well as the transportation of the extracellular ARG [56]. However, the introduction into the soil environment via manure fertilization has been reported to cause an alteration in phylogenetic structure, amplification in resistance level, and disturbance in ecological function (e.g., nutrient cycling) in the microenvironment [49,212,225]. 

Also, the degree or extent of the changes impacted on the soil will depend on the type of manure. In details, varying quantities of antibiotics are employed in livestock, and the quantity depends on the species of animals and the kind of farming system. The level of antibiotics found in manure available for a land application depends on manure management practices, which equally vary depending on the size of the herd, the type of livestock and farm operations, as well as the production stage of the animals [56]. According to Chee-Sanford et al. [56], the introduction of antibiotics in the soil may give a selective advantage to commensal bacteria harbouring resistance genes, or may create selective pressure, necessitating the acquisition of resistance genes in the commensal population. 

Numerous authors have characterized antibiotic-resistant bacteria and resistance genes from vegetables, including lettuce, cabbage, radish, green corn, onion, carrot, and fluted pumpkins grown on manure fertilized soils and irrigated with wastewater [97,223,226]. Specifically, Bonyadian et al. [227], in a study in Iran, demonstrated the presence of antibiotic-resistant verotoxigenic *E*. *coli* on vegetable samples collected randomly from retail shops. However, Kumar et al. [228] indicated that these vegetables absorb antibiotic contained in the manure. Therefore, the direct consumption by humans (uncooked or raw) of vegetables might lead to the transfer of antibiotic-resistant bacteria and their resistance genes, which may cause bacterial infections. Notwithstanding, Beuchat [229] and Johannessen et al. [230] suggested that the contamination of vegetable leaves may arise from diverse sources, such as manure, soil, water (for irrigation and cleaning), the handling equipment for product harvesting and processing, the mode of transportation, in addition to animals at both the pre- and post-harvest process. 

### 3.3. Antibiotic Resistance in the Water Environment

Microorganisms (specifically, bacteria) do not live in isolation [4], but are found in milieu/medium (humans, air, water, plants, and soil) known as their habitat (aquatic ecosystem), which offers them with the appropriate nutritional and growth requirements necessary for survival. Consequently, water represents one of the most important habitats for bacteria on the planet earth, and serves as a main natural route for the dissemination of microorganisms between different environmental compartments and/or aquatic ecosystems, humans, and other animals [231,232]. According to Taylor et al. [233], the aquatic environment is considered as a fundamental setting for environmental release, transformation, mixing, and persistence of antibiotic residues, antibiotic-resistant bacteria, and antibiotic resistance genes. The water (aquatic) environment can be subdivided into marine and fresh water based on salinity, average temperature, depth, and nutrient content [234]. More elaborately, the microbial aquatic environment includes surface and ground waters, drinking water, tap water, and wastewater. These waters have dynamic and distinct bacterial composition patterns influenced by temporal and spatial unevenness in physicochemical and biotic factors, including environmental stresses and nutrient composition [232,235]. Nevertheless, some known waterborne bacteria include *E. coli*, *Vibrio*, *Shigella*, and *Salmonella* species [236]. The aquatic environment has been reported to be the origin and reservoir of antibiotic-resistant bacteria and resistance genes [231,237].

Microbial communities respond to drastic changes in the ecosystem functioning, species composition and abundance, due to pollution [238,239], resulting in hypoxia, eutrophication, bioaccumulation and dissemination of pathogens [240]. Consequently, the abundance of antibiotic residues and pool of antibiotic-resistant bacteria and their resistance genes in aquatic ecosystems can be influenced or altered or amplified by the discharge of wastewaters from industrial and municipal wastewater treatment plants, runoff from manure-fertilized agricultural land, leakage from septic tanks and broken sewage pipes, and feces from wildlife, into water bodies [97,118,223,241]. 

Furthermore, the aquatic environments offer ecological and economic benefits [240]. Rivers serve as predominant sources of renewable water for freshwater ecosystems and humans, wherein they perform functions such as in irrigation, and are used as drinking water and for recreation [242]. Water is indispensable to life. Therefore, humans need clean and safe drinking water to protect their health from waterborne bacterial infections [236]. Water safe for human consumption needs to be free of pathogenic bacteria, although, the existence of pathogenic bacteria in water is intermittent, changeable, and at low levels, coupled with the fact that the isolation and cultivation of pathogenic bacteria from water sources are not straightforward. Therefore, routine microbiological water analysis does not involve the detection of pathogenic bacteria [236]. Interestingly, *Enterococci* and *E. coli* have been used in monitoring the fecal contamination of drinking and recreational water [235]. Nevertheless, fecal pollution of water sources is an increasing problem both in the developed and developing countries. In the latter countries, there may be an increase in the occurrence of pathogenic bacteria in river water near large urban populations, due to inept sewage treatment, fast-growing population, low income, and severe water stress. Hence, infection rates tend to be high, with waterborne pathogens [243]. Mulamattathil et al. [243] recovered coliforms and heterotrophic bacteria, as well as antibiotic-resistant *Aeromonas* and *Pseudomonas* species from surface and drinking water in Mafikeng, South Africa. The occurrence of these pathogens in drinking water might indicate serious health implications, especially among the immunocompromised individuals [244,245].

(a) Recreational waters and antibiotic resistance

Recreation has a pertinent role in the life of the human population, who always tend to couple the scene with water, be it man-made or natural. It has been reported that antibiotic resistant bacteria can equally be transmitted to humans during recreational use of fresh or marine water, which are natural aquatic habitats [246]. The natural aquatic environment, e.g., the surface waters, such as rivers and sea, serve as a receiving body of runoff water from farmlands fertilized with sewage sludge and animal manure, and wastewater discharged from treatment plants, which contain obvious concentrations of antibiotic resistant bacteria and their resistance determinants, along with biologically active metabolites of antibiotics or the parent compounds [247]. This causes great environmental damage and eutrophication, which might result in serious degradation of the water quality owing to the influx of high nutrients [248]. The quality of recreational water can be evaluated using fecal indicator bacteria, including *E. coli* and *Enterococcus*. Restricted to these two bacteria, the prevalence of antibiotic-resistant bacteria in the natural aquatic environment is always underestimated, because all the studies are geared towards measuring antibiotic resistance in the aforementioned culturable bacteria. However, other bacteria are present in the natural habitat, and might harbour antibiotic resistance genes which may equally contribute in the pool of resistance genes found in the habitat, thus, have the potential to disseminate bacterial resistance [249]. This is affirmed by the findings of Lihan et al. [250], who through culture-based methods and DNA fingerprinting unraveled the presence of single and multiple antibiotic-resistant *Enterobacter*, *Serratia*, *Pantoea*, *Klebsiella*, and *Citrobacter* in water samples obtained from a recreational river located in a resort community in Malaysia Borneo. Besides, the marine settings are special areas desired for recreational purposes and resting, and as such, are exposed to waste from residences, ships, and industries, as well as coastal areas which are site for highly populated cities, extensive fish farming, and recreational areas [251,252]. The transmission of the resistant bacteria and the resistance genes to humans depends on the type of water sport undertaken, as well as the density of the antibiotic-resistant bacteria in the water [249]. The concentration/magnitude of these resistant bacteria in the recreational waters is determined by the local landscape (e.g., coastal waters), wind speed, ultraviolet radiation, temperature, rainfall, and the source and level of pollution [253]. In a study conducted by Overbey and colleagues [247], higher levels of *E. coli* and *Enterococcus* were enumerated in recreational water samples collected from sewage-impacted sites in five beaches in Galápagos Island, Ecuador. In addition, Fernandes Cardoso de Oliveira et al. [254] revealed a higher level of multiple antibiotic resistance in heterotrophic marine bacteria recovered from sea water and sand in Gonzaguinha, the most organic polluted recreational beach, amongst Ilha Porchat and Guaraú beaches located in Southeast Brazil. 

Also, Alipour et al. [255] noted the presence of multiple antibiotic-resistant *Enterococcus* species characterized from the river and coastal water samples in the Northern part of Iran, therefore, these habitats are not suitable for swimming, as they would increase the risk of human exposure to antibiotic-resistant bacteria and resistance genes. Furthermore, Akanbi et al. [256] demonstrated the multiple antibiotic resistance patterns of *Staphylococcus aureus* isolated from recreational waters and beach sand identified by the occurrence of *mec* A, *fem* A, *rpo* B, *bla* Z, *erm* B, and *tet* M genes, in the Eastern Cape Province of South Africa. The beach water and sand can act as a plausible pool for antibiotic-resistant bacteria and resistance genes, and hence facilitate transmission via the direct ingestion of the seawater and/or direct contact with sea water during recreational activities undertaken in these waters [249]. In recent times, the awareness and involvement of many people in water sports have greatly increased [257], due to an increase in the local population and income earners, as well as tourism.

(b) Drinking water and antibiotic resistance

Most of the population in rural settings and urban settlements rely on untreated groundwater as a source of drinking water, due to the scarcity of fresh surface waters [258]. Untreated groundwater is considered safe for drinking, because it originates from the ground. Therefore, it is described as a natural water habitat protected from human intervention. Apparently, its microbiota reflects the natural population of the habitat [259]. Nevertheless, depending on the location/environment, groundwater becomes vulnerable to contamination with antibiotic residues, antibiotic resistant bacteria, and resistance genes from surface runoff of animal feces deposited on the ground in concentrated animal feeding operations, seepage of liquid/solid manure from storage sites (lagoons), leachate from landfill sites, spillage from broken sewage pipes, and leakage from septic tanks [56,118]. Also, in the North West province of South Africa, a study conducted by Carsten [260] revealed a very poor physicochemical and microbiological quality of water samples obtained from boreholes in the Mooi River and Harts River catchment areas. The authors attributed the results to the fecal contamination of groundwater with fecal bacteria and opportunistic bacteria that displayed different levels of antibiotic resistance.

Surprisingly, Kümmerer [118] reported that antibacterial agents found in groundwater are of considerable low concentration. In accordance with this, Li et al. [261] mentioned that the microbial concentration in groundwater is closely associated with its depth, season, weather conditions, and the type of adjacent land used in the concentrated animal feeding operations.

Wastewater constitutes “black water and grey water”. It describes all water with adversely affected quality due to influences from human activities [262]. Wastewater embodies water from livestock and poultry farms, aquaculture farms, and municipality that is sometimes released into the environment without treatment or used for irrigation by farmers involved in urban agriculture, since it contains significant amounts of micronutrients and organic matter, and provides water [263,264]. Due to the unavailability of good quality water worldwide, farmers resort to employing wastewater for irrigation, thereby promoting plant growth, water conservation, nutrient recycling, and reductions in inorganic fertilizer applied to the soil, and polluted water being discharged into the surroundings [265,266]. Owing to its numerous sources of water collection, wastewater contains diverse elements, inorganic mineral, antibiotic residues, antibiotic-resistant bacteria and resistance genes, human and animal feces and urine, etc. [267]. The sewage or wastewater treatment plant receives the wastewater, and partially treats or treats it, before discharge of its effluents into the environment. Therefore, the wastewater treatment plant serves as a link between human activities and the environment, and serves as a potential reservoir and release channel of antibiotic-resistant bacteria and resistance genes, giving a perfect opportunity for the transfer of antibiotic resistance genes [14,268,269]. The biological processes at the wastewater treatment plant might cause a reduction in the volume of antibiotics to varying degrees [267]. However, across the different regions of the world utilizing distinct types of wastewater treatment, these plants have been reported to be responsible for the discharge of about one billion cultivable coliform bacteria into the environment, indicating their inefficiency to reduce antibiotic-resistant bacteria and their resistance genes [270]. Clearly, Adefisoye and Okoh [268] demonstrated the occurrence of multidrug-resistant *E*. *coli* from the final discharged effluents of two wastewater treatment plants situated in the Eastern Cape Province, South Africa.

(c) Waterborne disease outbreaks caused by recreational/drinking waters versus antibiotic resistance

Outbreaks of diseases implicating water as a vehicle of transmission are termed waterborne disease outbreaks, because two or more persons are affected by a similar illness after being exposed to water, and are epidemiologically linked by time and by location of the water [271,272]. Humans become vulnerable to infections when exposed to the beaches when the waters are turbid and experiencing high waves, as well as after heavy rainfall; these cause the waters to be contaminated and unsafe [273]. Depending on the environmental conditions (climactic factors) and the characteristics of the pathogens, the waterborne pathogen grows in the aquatic habitat, however, its number, type, infectivity, and virulence is greatly influenced by the temperature, UV radiation, precipitation patterns, and water availability in the recreational waters [274]. 

According to Schets et al. [275], the waterborne illnesses in humans associated with the use of untreated or contaminated recreational water mainly comprise of gastroenteritis and skin infections. The authors further revealed that the bathing sites were predisposed to fecal contamination and environmental conditions that favoured the growth of naturally occurring pathogens. Children are viewed to be at a higher risk of gastroenteritis from recreational waters, because they last longer and swallow more water during swimming, as well as they play in shallow water and sand which are most contaminated, and experience a high tendency of hand to mouth exposure [273]. 

However, waterborne infections associated with exposure to drinking water include acute gastroenteritis, typhoid, diarrhoea, acute respiratory and neurological illness, and skin infections [272,276]. Between 2000 and 2001, South Africa witnessed a surprising and the biggest cholera outbreak in Africa, which involved 114,000 cases across nine provinces and caused 260 deaths, although it originated from KwaZulu Natal province [277]. Equally, Allam et al. [278] reported an outbreak of cholera involving 218 persons, which attacked a greater number of children (aged 5–14 years), males, and individuals greater than or equal to 60 years in Andhra Pradesh, India, and was caused by the contamination of reservoir water with *Vibrio cholerae*. This outbreak claimed three human lives. Even Biswas and colleagues [279] investigated an outbreak of cholera in Haibatpur village, India, caused by pond water contaminated with *Vibrio cholerae*. Ramurmutty and Sharma [280] suggested that the recurrent outbreaks of cholera in India are due to the presence of short-term carriers of *Vibro cholerae* in the communities, the constant change in biotypes and serotypes of this strain, development of resistance to the previous multiple antimicrobial agents used in the treatment of cholera- as well as poor water quality, unhygienic sanitation- and overcrowding. 

A shigellosis outbreak among 533 students in a rural elementary school in China was investigated, and findings were linked to the presence of *Shigella flexneri* 2b in untreated well water [281]. A diarrhoea outbreak in China caused by enteropathogenic and enterotoxigenic *E. coli* was noted among 131 individuals exposed to well water contaminated by river water [282]. Between May and August 2010 in Southern Vietnam, Nguyen and co-authors [283] documented an outbreak of diarrhea that infected seventy-one persons through the consumption of iced tea contaminated with *V. cholerae* O1, altered El Tor strains. Also, Ali et al. [284] noted a typhoid fever outbreak that affected 1430 individuals, originating from the consumption of fecally contaminated tap water both in military camps and the general population located in Kikwit, Democratic Republic of Congo, in 2011. In a Croatian study, Kovačić et al. [285] reported an outbreak of gastroenteritis, which affected 68 persons in a County Centre of Šibenik, caused by the pollution of groundwater used for drinking with *Salmonella enterica* subsp. *enterica* serovar *Enteritidis*. Fortunately, the isolates of the bacterium were susceptible to a range of antibiotics. 

Furthermore, Farooqui et al. [286] documented an outbreak of typhoid fever caused in Nek Muhammad village, Karachi—Pakistan, which involved over 300 hundred people, and resulted in the deaths of three humans in 2004. The findings in this study associated the outbreak to multidrug-resistant *Salmonella enterica* serovar Typhi ingested through well water that served as the sole source of drinking water in the community, but was unacceptably polluted with dead and decaying bodies of animal, fecal material, and garbage. Similarly, Ma et al. [287] reported the waterborne outbreak of shigellosis in China caused by *Shigella sonnei* with resistance to azithromycin, and third class cephalospsorins, due to *mph* (A) and *bla*_CTX-M-14_-harboring IncB/O/K/Z group transmissible plasmid. In addition, Akoachere et al. [288] unravelled the persistence of multidrug-resistant toxigenic *Vibrio cholerae* O1 in water samples (taps, dug wells, and streams) collected from New Bell—Doula, and associated these bacteria strains with the 2010–2011 cholera epidemic that recorded the highest number of death cases in the Littoral province of Cameroon.

In a nutshell, in developing countries, antibiotic-resistant bacteria are mostly found in animals, animal-derived food products, and agro-food environments [289]. Consequently, most of the gastrointestinal diseases of humans of public health concern including gastroenteritis, salmonellosis, shigellosis, cholera, listeriosis, campylobacteriosis, and yersiniosis are caused by pathogenic bacteria of public health and environmental importance via water and/or food contaminated with feces of animals or patients [236]. These diseases even become more complicated and severe with the presence of the antibiotic-resistant strains of the causative pathogens harbouring antibiotic resistance genes. As a result, Table 2 compiles information on the notorious environmental pathogens causing gastrointestinal and other diseases in humans/animals through the consumption of fecally-contaminated water and/or food, their resistance to antibiotics typical of human, veterinary and agricultural use, and the implicated antibiotic resistance genes occurring in some countries. 

The data in the tables represent the findings obtained from several studies conducted by different researchers across the world in their quest to determine antibiotic resistance genes in environmental sources, in a bid to strategically fight against the problem of rising antibiotic resistance. These findings were retrieved from published articles found in Science Direct, Pubmed, Medline databases, etc., using Google search engine. The search was done in a systematic way, and the search query term was modified using related terms to maximize accuracy. It can be depicted from Table 2, that antibiotic resistance is a global challenge, and needs to be addressed by the joint efforts from all the countries across the world, as we can never stop international travel and trade. Secondly, antibiotic resistant bacteria and resistance genes do not originate only from humans relating to clinical settings, but they are available and prevalent in the environment, food, and water that we consume. In addition, the table emphasises the presence of multidrug resistance in the respective countries, which is an uglier scenario, as the antibiotic resistance genes can be disseminated via food, water, and other environmental sources to clinical pathogens, thereby causing health threats to the public (especially the immunocompromised individuals who depend on antibiotics to boost their immune system). Consequently, owing to the high infectious disease burden in developing countries, alongside the poor sanitation and hygienic conditions, data in the tables create more awareness in the population about environmental antibiotic resistance, such that intervention measures be reinforced, to ensure prudent use of antibiotics, in a bid to contain antibiotic resistance. Furthermore, there is a clear need for continuous surveillance to monitor antibiotic resistance.

## 4. Conclusions

Antibiotics, naturally or semi-synthetically or synthetically produced substances, are very vital in the agricultural, veterinary, and clinical settings. These substances are endowed with bacteriostatic (inhibitory) or bactericidal activities necessary for prophylactic and therapeutic purposes in the lives of humans and animals, to prevent and treat diseases. Regardless of these beneficial roles, associated with antibiotics use are also hostile effects, in addition to their implementation at a sub-therapeutic level as growth promoters in feed and water consumed by livestock over an extended period. These may lead to antibiotic pollution, resulting in antibiotic residues in animal-derived products, including meat, milk, eggs, and edible tissues, and when consumed by humans, can cause direct toxicity, the development and emergence of antibiotic-resistant strains of bacteria, as well as therapeutic failure in clinical cases [136].

Of great concern are the food/waterborne pathogens responsible for life-threatening and difficult-to-treat gastrointestinal infections in humans, thus, of great concern to public healthcare systems worldwide. Nevertheless, following the chronicles of multidrug resistance of these pathogens in the developing countries, we hereby conclude that continuous surveillance of the antibiotic resistance profiles of bacterial pathogens (obtained from humans, animals, food, and other environmental sites) of public and environmental significance be implemented because of the high burden of infectious diseases. Also, patients harboring antibiotic-resistant infections might be unable to secure or afford effective second and third line antibiotic treatments. The situation is further compounded in these regions due to pollution of available water sources, poor hygienic measures, civil conflicts, increased population of immunocompromised individuals, lack of adequate laboratory infrastructures and personnel for the diagnosis of the disease-causing agents and surveillance of their antibiogram patterns, added to the deficiency in knowledge on prudent antibiotic use and antibiotic resistance [331].

## 5. Recommendations on Ways to Reduce the Overall Level of Antibiotic Resistance in Agricultural Settings

The unscrupulous use of antibiotics in farm animals has further compounded the problems of antibiotic resistance. Taking this into consideration, implementing measures that will help to reduce the abuse of antibiotics in livestock farming will, therefore, go a long way to further affect the level of antibiotic resistance. This is because there is an interrelationship between the environment and animals, which ultimately influences public health (humans). Altogether, the following recommendations are set forth; following the chronicles of the information studied, there is a general weakness among farmers regarding the lack of knowledge on antibiotic use and storage, its abuse, and consequent hazards to public health and the environment [32]. Accordingly, a sensitization campaign is inevitable to educate both the farmers and the general population on the dangers of the imprudent use of antibiotics, improper storage of antibiotics, and the resulting antibiotic resistance, with its consequences on public health. Also, veterinary activities and therapy are mostly assumed and performed by owners in industrial and semi-industrial production systems, regardless of whether they are qualified or trained [43]. Sekyere [41] equally highlighted that the veterinarian services are lacking due to the locations of the farms and/or the veterinarians are limited in number and their facilities. Notwithstanding, livestock farming contributes to the economy of the country, therefore, at this juncture, we hereby propose that the government and well renowned and financially viable private companies that are interested in the economic development of the country should encourage the employment of regular, proper, and efficient veterinarian services via subsidies to farmers, especially to lower-income farmers in the rural and remote settings of the country.

Furthermore, the indiscriminate use of antibiotics (that is, without prescription, supervision, and guidance from veterinarians) in animal farming should be avoided. Veterinary officers and pharmacists should adhere strictly to the policies governing prescriptions of antibiotics used in animal farming. They should equally ensure (via sensitization and consultation) that the farmers adhere to the dosage, length, and administration of treatment and withdrawal periods of antibiotics prescribed for a purpose, as well as the causative agent of the disease be diagnosed and antibiotic susceptibility testing conducted against the disease causative agent prior to prescription and employment of the antibiotics [41,43]. Moreover, routine surveillance and detailed analysis of antibiotic residues present in foods of animal origin should be conducted before being dispatched or delivered for human consumption. Owing to the variations in antibiotic consumption patterns and differences in animal production systems between countries [12,43], we therefore advise that based on the country, stringent control and/or regulations should be implemented by policy makers to enforce the legitimate purchase and employment of antibiotics in animal farming. 

Nonetheless, farmers should be educated on the need of good and proper hygienic/sanitation conditions and facilities which nurture healthy living in the animals and preclude the opportunities for the emergence of disease conditions and infections, thus evading therapeutic antibiotic regimens. Ultimately, the use of antibiotics in food-producing animals should be limited by incorporating possible alternative substances, including probiotics, prebiotics, and plant-derived or crude plant extracts for the treatment and prevention of diseases [9,25]. Consequently, persistent and extensive surveillance should be conducted by the government in collaboration with the public health authorities to monitor antibiotic consumption in animal farming, in addition to the antibiotic resistance profiles of notorious foodborne pathogens to well-known drugs used in animal farming. Furthermore, the government and public health sector should make available, services for research and analysis of data obtained via the surveillance studies in order to track the drug and its residue level in most environmental samples.

## Figures and Tables

**Table 1:** Presence of varying concentrations of antibiotic residues in the different animal-derived products in some developing countries.

Antibiotic Residue	Concentration	Sample	Consequences in Humans/Animals	Country	Literature
Oxytetracycline		Chicken	Carcinogenicity, cytotoxicity in the bones of broiler chickens. Presence of residues cause technological challenges during milk processing.	Tanzania	Kimeria et al. [85]
	2604.1 ± 703.7 μg/kg	Muscle			
	3434.4 ± 604.4 μg/kg	Liver			
	3533.1 ± 803.6 μg/kg	kidney			
		Beef		Nigeria	Olufemi and Agboola [76]
	51.8 ± 90.53 μg/kg	Muscle			
	372.7 ± 366.8 μg/kg	Kidney			
	1197.7± 718.9 μg/kg	Liver			
		Cattle		Ethiopia	Bedada et al. [86]
	15.92 to 108.34 μg/kg	Muscle			
	99.02 to 112.53 μg/kg	kidney			
Enrofloxacin	0.73 and 2.57 μg/kg	Chicken tissues	Allergic hypersensitivity reactions or toxic effects, phototoxic skin reactions, chondrotoxic), and tendon rupture	Iran	Tavakoli et al. [87]
Chloramphenicol	1.34 and 13.9 μg/kg		Bone marrow toxicity, optic neuropathy, brain abscess		
Penicillin	0.87 and 1.3 μg/kg	Calves muscles	Allergy, affect starter cultures to produce fermented milk product		
Oxytetracycline	3.5 and 4.61 μg/kg		Carcinogenicity, cytotoxicity in the bones of broiler chickens		
Quinolones	30.81 ± 0.45 μg/kg	Chicken	Allergic hypersensitivity reactions or toxic effects (phototoxic skin reactions, chondrotoxic) and tendon rupture	Turkey	Er et al. [88]
	6.64 ± 1.11μg/kg	Beef			
Tetracyclines		Chicken	Primary and permanent teeth discolouration in children and infants, allergic reactions and teratogenicity during the first trimester of pregnancy, nephrotoxicity, carcinogenic, hepatoxocity, and disturbance of the normal microflora of the intestines. It equally causes skin hyperpigmentation of areas exposed to the sun, proximal and distal renal tubular acidosis, hypersensitivity reactions	Egypt	Salama et al. [89]
	124 to 5812 μg/kg	Breast			
	107–6010 μg/kg	Thigh			
	103 to 8148 μg/kg	Livers			
		Chicken		Cameroon	Guetiya-Wadoum et al. [25]
	150 ± 30 μg/g	Liver			
	62.4 ± 15.3 μg/g	muscle			
		Beef		Kenya	Muriuki et al. [90]
	50 to 845μg/kg	Kidney			
	50 to 573 μg/kg	Liver			
	23–560 μg/kg	muscles			
Amoxicillin	9.8 to 56.16 μg/mL	Milk	Carcinogenic, teratogenic, and mutagenic effects	Bangladesh	Chowdhury et al. [91]
	10.46 to 48.8 μg/g	Eggs			
Sulfonamides	16.28μg/kg	Raw milk	Carcinogenicity, allergic reactions	China	Zheng et al. [92]
Quinolones	23.25 μg/kg		Allergic hypersensitivity reactions or toxic effects (phototoxic skin reactions, chondrotoxic) and tendon rupture		
Oxytetracycline	199.6 ± 46 ng/g	Beef	Carcinogenicity, allergic reactions	Zambia	Nchima et al. [93]
Sulphamethazine	86.5 ± 8.7 ng/g				
Penicillin G	15.22 ± 0.61 μg/L	Fresh milk	Allergy (hypersensitivity reaction) ranging from mild skin rash to life-threatening anaphylaxis	Nigeria	Olatoye et al. [94]
	7.60 ± 0.60 μg/L	Cheese (wara)			
	8.24 ± 0.50 μg/L	Fermented milk (nono)			
Sulphonamides		Chicken	Carcinogenic potential and mild skin rash to severe toxiderma, epidermal toxic necrolysis, blood dyscrasias	Malaysia	Cheong et al. [95]
	0.08–0.193 μg/g	Liver			
	0.006–0.062 μg/g	Breast			
Tetracycline	>0.1 μg/mL	Raw milk	Primary and permanent discolouration of teeth in children and infants, teratogenicity during the first trimester in pregnancy, etc.	India	Nirala et al. [96]
Oxytetracycline			Carcinogenicity and cytotoxicity in bone marrow of broiler chickens		
Sulfadimidine			Carcinogenicity and allergic reactions		
Sulfamethoxazole					

**Table 2:** Notorious pathogens, antibiotic resistance, and implicated resistance genes from environmental samples in some developing countries.

Pathogens	Antibiotic Resistance	Resistance Genes	Source	Diseases Caused/Symptoms	Country	Literature
*Escherichia coli*	Multidrug resistance	*bla*_CTX-M-9_, *qnr* S	Drinking water	Diarrhoea, septicaemia, urinary tract infections, neonatal meningitis, abdominal pain, fever, pneumonia, hemolytic uraemic syndrome, nosocomial bacteraemia	India	Purohit et al. [290]
		*bla*_CTX_, *bla*_TEM_	Vegetables, raw eggs, raw chicken, unpasteurized milk, raw meat			Rasheed et al. [105]
		*bla*_CTX-M-15_, *bla*_OXA-1_, *bla*_CMY-2_, *qnr* S, *qnr* B	Household water supply		Bangladesh	Talukdah et al. [291]
		NA	Bird cloacae		Saudi Arabia	Shobrak and Abo-Amer [206]
		*bla*_CTX_, *bla*_SHV_, *bla*_TEM_, *bla*_CTX-M_ + *bla*_TEM_, *bla*_CTX-M_ + *bla*_SHV_, *bla*_CTXM_ + *bla*_SHV_ + *bla*_TEM_, *bla*_TEM_ + *bla*_SHV_	Poultry swab		Zambia	Chishimba et al. [292]
		*tet*, *dfr* A, *bla*_TEM-1_	Animal feces		Zimbabwe	Mercat et al. [293]
		*Sul* I, *Sul* II, *bla* PSE1, *Amp*C	Zoo lake		Brazil	De Faria et al. [294]
		NA	Animal feces		Zambia	Mainda et al. [295]
		*bla*_TEM_, *bla*_CMY-2_	Milk, water		Thailand	Hinthong et al. [296]
		NA	Drinking water		Ghana	Adzitey et al. [297]
*Escherichia coli*	Multidrug resistance	*bla*_TEM_, *bla*_OXA-1_, *tet* A, *Sul* 1, *Sul* 2, *Sul* 3, *dfr*A1-*Sat*1-*aad*A1-*orf*X gene cassette, *Sat*-*psp*-*aad*A2-*cml*A1-*caad*A1-*qa*cH-*IS*440-*Sul*3	Chicken and turkey meat	Diarrhoea, septicaemia, urinary tract infections, neonatal meningitis, abdominal pain, fever, pneumonia, hemolytic uraemic syndrome, nosocomial bacteraemia	Tunisia	Soufi et al. [298]
		NA	Curds, pasteurized milk, déguè		Burkina Faso	Bagré et al. [299]
		*bla*_TEM-1_, *bla*_CTX-M_, *bla*_SHV_, *tet* A, *tet* B	Drinking water,		Tanzania	Lyimo et al. [300]
		*bla*_TEM_, *Sul* 2, *Sul* 3, *aad* A, *str* A, *str* B, *cat* A1, *cml* A1, *tet* A, *tet* B, *qnr*S	Waste, litter, soil and water samples from poultry farms		Nigeria	Adewolo et al. [301]
		NA	Vegetables		Iran	Bonyadian et al. [227]
		*bla*_CTX-M_, *bla*_TEM-1_	Soil, feces		China	Gao et al. [108]
		*bla*_CTX-M_, DHA-1(*Amp*C)	Retail food Pearl river			Ye et al. [302]
		*Sstr* A, *aad* A, *cat 1*, *bla*_TEM_, *tet* A, *tet* B, *tet* C, *tet* D, *tet* K, *tet* M	Effluent from wastewater treatment facilities		South Africa	Adefisoye and Okoh [268]
*Salmonella* sp.	Multidrug resistance	*tet* A, *Sul* 1	Water, soil, meat, vegetables, dry foods	Salmonellosis (diarrheal disease), bacteraemia	Kenya	Christabel et al. [303]
*Escherichia coli*						
*Shigella* sp.				Shigellosis (bacillary dysentery)		
*Salmonella enteritidis*	Multidrug resistance	NA	Food	Salmonellosis, bacteraemia	Brazil	Oliveira et al. [304]
*Salmonella typhimurium*	Multidrug resistance	*bla*_TEM_, *tet* A, *tet* C, *Sul* 1, *Sul* 3, *cat* 1, *flo* R, *dfr*A12, *orf* F, *aad* A2	Chicken (liver, intestinal contents, gall bladder	Salmonellosis, bacteraemia	Egypt	El-Sharkawy et al. [305]
*Staphylococcus aureus*	Multidrug resistance	*mec* A, *bla* Z, *tet* K	Broiler chicken (caecum, feces, retail meat	Bovine mastitis, endocarditis, osteomyelitis, furunculosis, necrotizing pneumonia, toxin syndromes, gastroenteritis, abscess formation	South Africa	Mkize et al. [306]
		NA	Milk			Ateba et al. [307]
*Salmonella enterica*	Multidrug resistance	NA	Feces of poultry, swine and hedgedogs	Salmonellosis (diarrhoeal disease), bacteraemia	Burkina Faso	Kagambèga et al. [130]
*Salmonella enterica* subsp. *enterica*			Food, water, and animal		Sudan	Elmadiena et al. [308]
*Enterococcus faecium*	Multidrug resistance	*Tet* M, *tet* L, *erm* B	Chicken feces and residual water	Neonatal meningitis, vancomycin-resistant enterococci (VRE) infections, nosocomial infections	Angola	Martins et al. [309]
*Vibrio* species	Multidrug resistance	*bla*_P1_, *dfr* A15, *aad* A2 cassettes	Surface urban water, shell fish	Diarrhoea, cholera/profuse watery stool (rice water stool)	Mozambique	Taviani et al. [310]
*Enterococcus* species	Multidrug resistance	*van*A, *van*C1, *vanc*2	Meat, fish, vegeatbles, pasteurized milk, cheese	Nosocomial infections, neonatal meningitis, urinary and wound infections	Egypt	Raafat et al. [311]
		*erm* B, *tet* M-*tet*K, *aph*(3′)-III, *ant*(6), *van* C2, *aac*(6′)-*aph*(2′′)	Vegetables		Tunisia	Said et al. [312]
						Said et al. [313]
		*tet* M, *tet* L, *erm* B, *cat* gene, *aph*(3)-IIa gene, *aac*(6′)-*aph*(2′′) and *ant*(6)-1a	Seafoods			
*Staphylococcus* sp.	Multidrug resistance	NA	Starch, meat, salad, vegetables	Skin infections, bovine mastitis, infections associated with prosthetic devices and catheters, gastroenteritis, abscess formation	Botswana	Loeto et al. [314]
		*bla* Z, *tet* M	Milk and traditional cheese and kashk		Iran	Jamali et al. [315]
*Campylobacter* sp.	Multidrug resistance	NA	Chickens	Campylobacteriosis, post infectious irritable bowel syndrome	South Africa	Bester and Essack [33]
*Pseudomonas* sp.	Multidrug resistance	*tet* A, *Sul* 1, bla _TEM_, (*aad*A2, *aad*A1), *dfr* A15, *dfr* 1	Treated and untreated water	Infections of the blood, bone, urinary tract, central nervous system, and wounds endocarditis, pneumonia (nosocomial infections)	Nigeria	Adesoji et al. [316] Igbinosa and Obuekwe [317]
*P. aeruginosa*	Multidrug resistance	*bla*_OXA_, *bla*_IMP_, *bla*_AMPC_, *bla*_TEM_, *tet* C	Abattoir environment			
*Salmonella* sp.	Multidrug resistance	NA	Meat, meat product	Salmonellosis (diarrheal disease), bacteraemia	Uganda	Bosco et al. [211]
		*pse*-1, *tet* A, *ant*(3′′)-1a, *tet* B *tet* A, *Sul*1, *Sul*2, *ant*(3′′)-1a, *pse*-1, *tet* B	Broiler’s chicken (South Africa) Broiler’s chicken (Brazil)		South Africa	Zishiri et al. [318]
		NA	Raw meat, minced meat, burger samples, raw eggs, raw milk		Ethiopia	Ejo et al. [104]
		*dfr*A12-*aad*A2, *aad*A2-*lin* G, *qnr* S	Pigs, retail pork		Thailand & Laos	Sinwat et al. [319]
		NA	Raw chicken		Senegal	Bada-Alambedji et al. [320]
		NA	Beef		Namibia	Shilangale [321]
*Salmonella* sp.	Multidrug resistance	NA	Street foods including macaroni, salad, bean cooked in sauce, rice in sauce	As indicated above	Benin Republic	Sina et al. [322]
*Staphylococcus aureus*						
*Escherichia coli*	Multidrug resistance	NA	Turkey meat	As indicated above	Morocco	Abdellah et al. [323]
*Staphylococcus aureus*						
*Aeromonas* sp.	Multidrug resistance	NA	Surface water, wastewater effluent	Gastroenteritis and wound infections	South Africa	Olaniran et al. [324]
*Listeria* sp.				Listeriosis		
*Aeromonas* sp.	Multidrug resistance	NA	Surface water, drinking water	As indicated above		Mulamattathil et al. [243]
*Pseudomonas* sp.				As indicated above		
*Salmonella typhimurium*	Multidrug resistance	NA	Animal feces, eggs	Salmonellosis(diarrheal disease), bacteraemia	Kenya	Nyabundi et al. [325]
*Salmonella enteritidis*						
*Escherichia coli*	Multidrug resistance	*bla*_TEM_, *aad*A, *bla*_CTX-M_, *bla*_SHV_	Hospital effluent, wastewater, urban rivers	As indicated above	Democratic Republic of Congo	Laffite et al. [326]
*Enterococcus* sp.						
*Pseudomonas* sp.						
*Bacillus* sp.	Multidrug resistance	NA	Oluwa river	Meningitis, anthrax, pneumonia, food poisoning	Nigeria	Ayandiran et al. [327]
*Staphylococcus* sp.						
*Streptococcus* sp.						
*Pseudomonas* sp.				As indicated above		
*Micrococcus* sp.				Pulmonary infection, septic shock, recurrent bacteraemia		
*Klebsiella oxytoca*	Multidrug resistance	NA	Surface water	Septic shock, urinary tract infection	Mexico	Delgado-Gardèa et al. [328]
*Escherichia coli*				As indicated above		
*Enterobacter cloacae*				Intra-abdominal tract infections, septic arthritis, ophthalmic infections, bacteraemia, UTI		
*Enterobacter cloacae*	Multidrug resistance	*dfr* A1, *dfr* A5, *dfr* A7, *dfr* A12, *dfr* A15, *dfr* A17, *dfr* A25, *aad* A1, *aad* A2, *aad* A5, *aad* A7, *aad* A12, *aad* A22, *aac*(3)-Id, *cml* A, *ere* A2, *arr*3, *bla*_TEM_, *bla*_SHV_, *bla*_CTX-M_, *bla*_OXA_, *flo* R, *qnr* S, *aac*(6)-Id-cr	Milk	Bovine mastitis, pulmonary pneumonia, septic shock, nosocomial infections, urinary tract infections, septicaemia, wound infection, bacteraemia, hepatic, pancreatic and biliary disease, nosocomial inefctions (UTI, wounds, blood and lower respiratory tract	Egypt	Ahmed and Shimamoto [329]
*Klebsiella pneumoniae*						
*Klebsiella oxytoca*						
*Escherichia coli*						
*Citrobacter freundii*						
*Citrobacter* sp.	Multidrug resistance	*bla*_TEM_, *tet* A, *mef* A, *erm* B, *Sul* 1, *int* 1	Drinking water	Variety of nosocomial infections	Uganda	Soge et al. [330]
*Escherichia coli*				As indicated above		
*Enterobacter* spp.						
*Klebsiella* sp.						
*Morgnella morganii*				Neonatal sepsis, arthritis, UTI		
*Proteus* spp.				UTI and formation of stones		
*Providencia rettgeri*				Cystitis, complicated UTI		
*Pseudomonas* sp.				As indicated above		
*Salmonella* sp.						
*Serratia odorifera*				Not involved in any invasive disease		
